# Unconventional Hysteretic Charge Filling in Moiré‐Reconstructed Helical Trilayer Graphene

**DOI:** 10.1002/advs.75919

**Published:** 2026-06-01

**Authors:** Hangyeol Park, Junhyeok Oh, Rasoul Ghadimi, Chiranjit Mondal, Yungi Jeong, Won Beom Choi, Kenji Watanabe, Takashi Taniguchi, Bohm‐Jung Yang, Joonho Jang

**Affiliations:** ^1^ Department of Physics and Astronomy Seoul National University Seoul South Korea; ^2^ Institute of Applied Physics Seoul National University Seoul South Korea; ^3^ Center for Theoretical Physics (CTP) Seoul National University Seoul South Korea; ^4^ Research Center for Electronic and Optical Materials National Institute for Materials Science Tsukuba Japan; ^5^ Research Center for Materials Nanoarchitectonics National Institute for Materials Science Tsukuba Japan

**Keywords:** anderson localization, condensed matter physics, electronic band structure, hysteresis, moiré pattern, quantum state, quantum, superlattice, van der Waals heterostructures

## Abstract

Moiré superlattices in van der Waals heterostructures are powerful tuning knobs for engineering electronic band structures and emergent phases. Expanding this framework to multi‐moiré architectures enables the discovery of novel quantum states, as hierarchically superposed patterns generate a versatile and sophisticated electronic landscape. Moreover, competition between interlayer interactions and lattice strain can drive structural reconfigurations, forming periodic domains delineated by aperiodic boundaries. Here, anomalous hysteretic charge filling is observed in helical trilayer graphene, where sequential twist angles create a complex moiré‐of‐moiré environment. Transport measurements reveal dual electronic signatures: moiré‐induced minibands in periodic domains, and a distinct hysteretic resistance signal from the aperiodic boundaries. These observations can be interpreted within the framework of partial electron localization driven by the loss of global periodicity in the incommensurate regime. This work provides insights into how spatially inhomogeneous potentials reshape electronic states and establishes a pathway for controlling quantum phases in non‐periodic lattice environments.

## Graphical abstract

In helical trilayer graphene, sequential twisting reconstructs the moiré landscape into periodic domains separated by aperiodic boundaries. Longitudinal transport reveals sweep‐direction‐dependent hysteresis, while the Hall response traces this behavior to hysteretic charge filling at the aperiodic boundaries. These results offer insight into how spatially inhomogeneous potentials reshape electronic states in multi‐moiré systems.

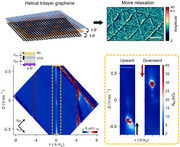

## 1 Introduction

For decades, the exploration of novel electronic states was primarily constrained by the fixed symmetries and chemical compositions of naturally occurring crystals [1, 2]. The emergence of van der Waals (vdW) heterostructures has transcended these limitations, inaugurating an era where two‐dimensional (2D) electronic systems are no longer merely discovered, but purposefully engineered at the nanoscale [3, 4, 5]. By leveraging the subtle interference between stacked and twisted atomic layers, these platforms enable the realization of moiré superlattices [6]—synthetic periodicities that serve as a versatile template for tailoring electronic potential landscapes. This unprecedented structural control allows for a profound reconfiguration of the underlying band structure, unveiling a rich spectrum of emergent phenomena spanning correlated insulators [7, 8, 9], superconductivity [9, 10, 11, 12, 13], topological insulators [14, 15, 16, 17, 18, 19, 20], and ferroelectricity [21, 22]. The remarkable success of these systems has established them not only as a preeminent platform for exploring intrinsic quantum interactions but also as a transformative framework for next‐generation device science, where the electronic properties of 2D electron systems are precisely dictated by intentional structural design.

While significant strides have been made in single‐moiré architectures, a more intricate electronic environment emerges when multiple interference patterns are superimposed within a single heterostructure. This moiré‐of‐moiré—or supermoiré—structure introduces an additional hierarchy of length scales and potential modulations that lie beyond the reach of conventional periodic lattices [23, 24, 25]. What makes these multi‐moiré systems even more intriguing is their capacity to host emergent superstructures through moiré relaxation, which further modifies the electronic spectra by favoring interlayer commensurability over intralayer distortion [26, 27, 28, 29, 30, 31, 32, 33, 34, 35, 36]. Notably, by tuning the twist angles between layers, this relaxation can lead to a remarkable configuration characterized by commensurate moiré domains surrounded by aperiodic, incommensurate domain boundaries [32, 33, 34, 35, 36]. Such features facilitate the investigation of electronic phases within incommensurate regimes—regions where the lack of global periodicity transcends the long‐standing paradigms of the Bloch limit. By moving beyond the constraints of traditional crystalline symmetry, these aperiodic landscapes offer a fertile ground for discovering novel quantum phases that are fundamentally inaccessible in periodic systems.

In this context, twisted three‐layer graphene represents the simplest platform for achieving the moiré domain structure, as it naturally hosts two moiré superlattices generated by sequential twisting. Here, we investigate helical trilayer graphene (hTG), a system consisting of three monolayer graphene sheets stacked sequentially with two equal twist angles. Strikingly, we observe hysteretic resistance responses across multiple devices, which we attribute to unusual transport phenomena along domain boundaries—regions with aperiodic lattice structures that emerge between adjacent moiré domains. Upon further investigation, we strongly suggest that hysteretic charge filling within these areas underlies the observed transport anomalies by modulating the carrier density participating in transport.

## 2 Results

### 2.1 Lattice Relaxation and Characterization of Helical Trilayer Graphene

**FIGURE 1:**
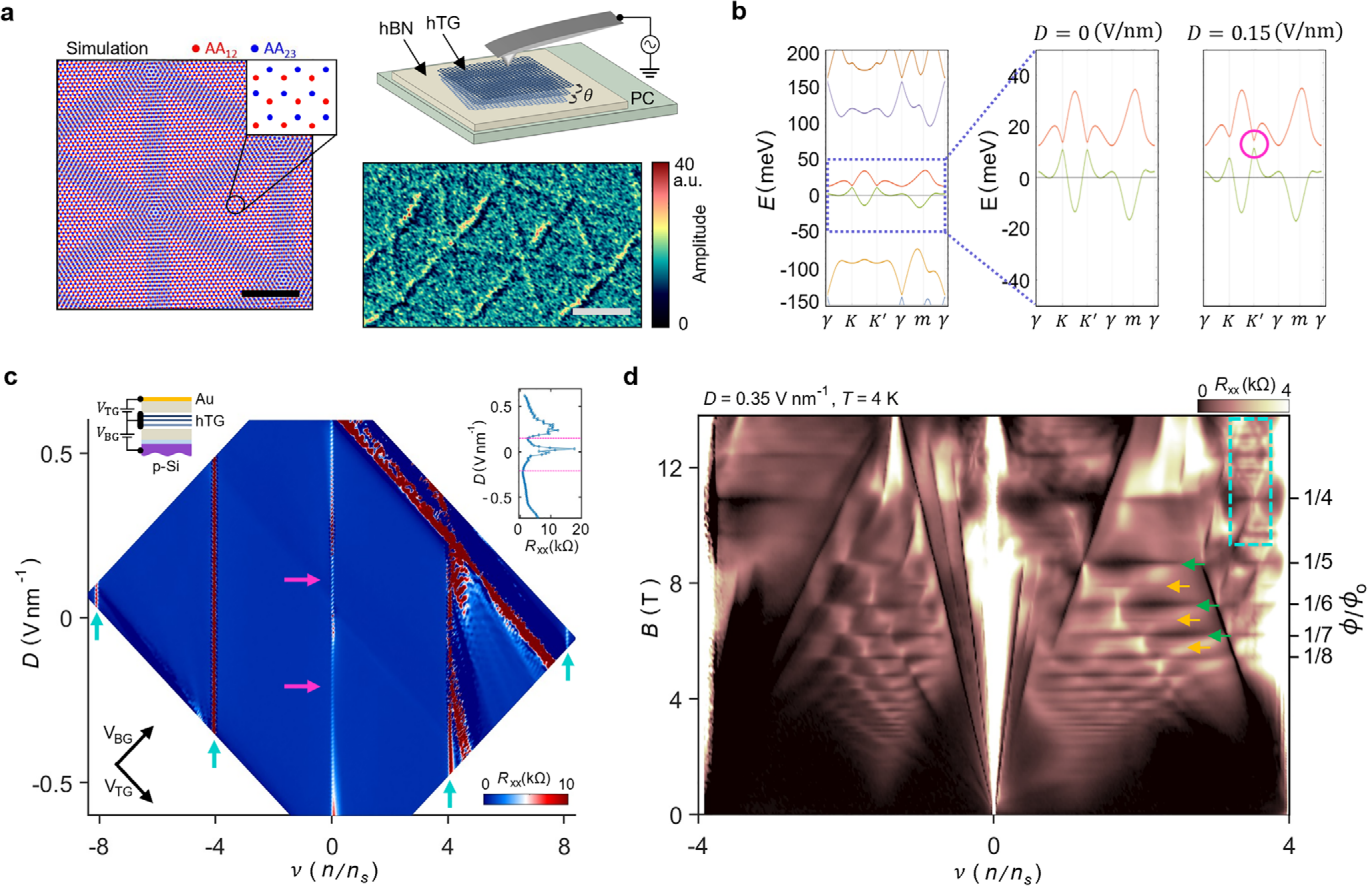
Lattice relaxation and characterization of hTG. (a) (Left) Lattice configuration obtained from LAMMPS simulations of hTG at *θ* = 1.8°, highlighting the effects of moiré relaxation (scale bar: 100 nm). Blue and red dots indicate the AA sites of the upper and lower moiré patterns, respectively. Atomic reconstruction leads to the formation of periodic domains separated by aperiodic domain boundaries, emphasized by shaded blue lines. The inset shows a magnified view of a representative periodic domain. (Right) LPFM measurement is performed at an intermediate stage of the fabrication process. A schematic of the measurement configuration is shown at the top, and the corresponding LPFM amplitude map is shown below (scale bar: 200 nm). Domain‐wall structures associated with lattice relaxation are visible in the map. (b) (Left) Electronic band structure at the K valley of a single domain in *θ* = 1.35° hTG at *D* = 0 V nm^−1^, with each isolated band shown in a distinct color. (Center) Zoom‐in of the region near the charge neutrality point (CNP), as indicated by the purple dashed box in the left panel. (Right) Band structure at *D* = 0.3 V nm^−1^ after the gap reopening. The pink circle marks the band‐touching point encountered upon increasing *D*. (c) Longitudinal resistance map as a function of *n* and *D* at T = 2.5 K for the hTG1 device with a twist angle of 1.35^○^. The data show resistance peaks at the CNP and at moiré band insulators (ν = ±4, ±8) as indicated by cyan arrows. At the CNP, two local resistance minima are highlighted by pink arrows. The upper‐left inset shows a schematic of the measurement configuration, and the upper‐right inset presents a line‐cut plot at the CNP, with two pink dotted lines marking the positions indicated by the pink arrows in the main panel. (d) Landau fan diagram at *D* = 0.35 V nm^−1^ and T = 4 K for the same device. The right y‐axis represents the magnetic field normalized by the magnetic flux quantum. The green and orange arrows indicate the first‐ and second‐order BZ oscillations, respectively, while higher‐order BZ features appear within the cyan dotted box.

We fabricated hBN‐encapsulated hTG devices with dual gates (see Experimental Section), allowing control over both electronic density (*n*) and displacement field (*D*), with various twist angles (*θ*). Without lattice relaxation, the two moiré patterns—formed between the top and middle graphene layers, and between the middle and bottom layers—are rotated by the same twist angle *θ*. They are thus expected to generate a supermoiré pattern, so‐called moiré‐of‐moiré superstructure, whose characteristic length scale λ_*sm*_ = λ_*m*_ /2sin(θ/2) is significantly larger than the original moiré period λ_*m*_ =  *a*/2sin(θ/2), where *a* denotes the graphene lattice constant. However, it has been proposed that when the twist angle falls within a moderate range of approximately 1° to 3°, moiré lattice relaxation gives rise to triangular domains featuring a periodic arrangement of moiré sites (red and blue dots in the left panel of Figure 1a), separated by domain boundaries where moiré sites appear at aperiodic positions [32, 35]. Direct experimental evidence for moiré lattice relaxation is provided by lateral piezoresponse force microscopy (LPFM) characterization performed at an intermediate stage of device fabrication, where the stamp‐supported hBN–hTG assembly was inverted and probed prior to final device assembly (right panel of Figure 1a; see Experimental Section). The LPFM amplitude map resolves triangular domain‐wall networks, consistent with the formation of relaxation‐induced domains and complementing earlier theoretical predictions [30] and scanning probe studies [33].

The periodicity of the domains allows their electronic band structure to be computed within the continuum approximation (see Experimental Section). As an example, the calculated band structure for a single K valley at *D* = 0 V nm^−1^ is presented in Figure 1b. It shows that energy bands near the charge neutrality point (CNP) are well‐separated by energy gaps reaching up to several tens of meV, and accommodate four electrons per moiré unit cell (Figure S1). Importantly, as the *D* field increases (or decreases), the energy gap between the two isolated bands near the CNP gradually closes—leading to a band touching (at the point indicated by the pink circle in Figure 1b for the increasing‐*D* case)—and then reopens (see Section S3). Such theoretical predictions are well reflected in our transport measurements. Figure 1c shows the measured four‐probe longitudinal resistance map along the *n*–*D* plane for the hTG1 device with a twist angle of 1.35°. The high‐resistance vertical lines, indicated by cyan arrows, correspond to the moiré fillings at 𝜈 = 0, ±4 and ±8, in agreement with the calculated band structure. A closer examination of the resistance at the CNP reveals two local minima as the *D* field varies as indicated by pink arrows (see also inset for the line‐cut plot), consistent with the presence of two band inversion points.

We also performed magnetotransport measurements. As shown in Figure 1d, the magnetoresistance at *D* = 0.35 V nm^−1^ and T = 4 K exhibits well‐developed Landau fan features, along with Brown–Zak (BZ) oscillations persisting up to the fifth order (cyan dashed box and Figure S2). Clear Landau fan features reflect the high quality of the device, and the appearance of BZ oscillations is consistent with the underlying moiré periodicity inside the domains. In addition, key signatures—including high‐resistance states appearing at moiré fillings that are multiples of four, and BZ oscillations—are reproducibly observed across multiple devices fabricated under comparable conditions, supporting that the phenomena reported in this work are intrinsic rather than device‐specific artifacts (Section S2).

### 2.2 Hysteretic Transport in hTG

**FIGURE 2:**
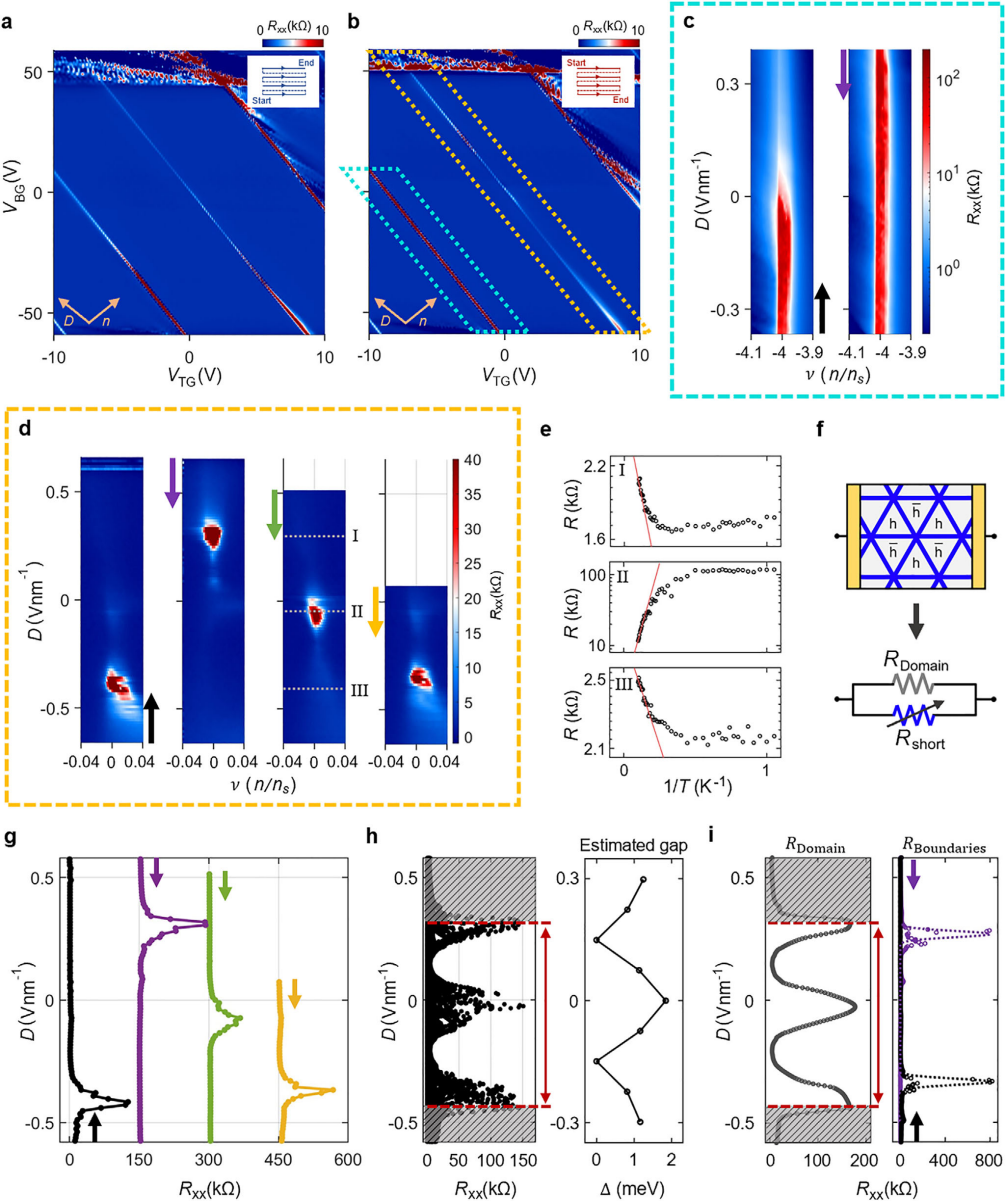
Hysteretic responses of total resistance. (a, b) Longitudinal resistance maps as a function of *V*_TG_ and *V*_BG_, measured during upward (a) and downward (b) sweeps of *V*_BG_. In both maps, *V*_TG_ was swept as the fast axis and *V*_BG_ as the slow axis. High‐resistance regions near ν = −4 (cyan dotted box) and 0 (orange dotted box) exhibit clear hysteresis depending on the sweep direction. Insets show arrows indicating the direction of the gate sweeps. (c) High‐resolution resistance maps near ν = −4 (corresponding to the cyan dotted box in (b)), replotted as a function of *n* and *D*. These data were acquired during full‐range dual‐gate sweeps. (d) High‐resolution resistance measurements near the CNP were obtained under varying sweep conditions. The first two panels were acquired during full‐range dual‐gate sweeps, while the third and fourth panels were obtained with reduced slow axis (*V*_BG_) ranges ([−60 to 6 V] and [−60 to 42 V], respectively). (e) Arrhenius plots of *R*_xx_ near the CNP at *D* = 0.3 V nm^−1^ (I), −0.1 V nm^−1^ (II) and −0.4 V nm^−1^ (III). All data were acquired under conditions where the resistive packet was positioned near *D* = 0, as shown in the third panel of (D). (II) exhibits insulating behavior, whereas (I) and (III) show metallic behavior. (f) Schematics of the toy model, represented as parallel‐connected circuits, were used to interpret the observed hysteresis. *R*_Domain_ and *R*_Short_ represent the resistance of the domains and domain boundaries, respectively. (g) Device resistances at the CNP, extracted as line‐cuts from the dual‐gate maps in (d). (h) (Left) Resistance profile compiled from an extensive set of gate‐sweeps with varying resistive packet positions. The envelope in the unshaded region between the red dashed lines reflects the intrinsic domain resistance. (Right) Calculated evolution of the energy gap at the CNP as a function of *D*, showing gap closing and reopening consistent with the experimental envelope. (i) Extracted *R*_Domain_ at the CNP (left) and that of the domain boundaries (right). In the right panel, black and purple traces show representative upward and downward sweeps, respectively.

While basic features in the measurement data of Figure 1 are well accounted for by the calculations, in Figure 2, we observed a peculiar but distinct hysteretic behavior, where the measured resistance values significantly changed depending on the sweep directions of the gate voltages. First, a resistance map measured as a function of the bottom gate voltage *V*_BG_ (serving as the slow axis of 2D voltage sweeps, from −60 to 60 V) and the top gate voltage *V*_TG_ (serving as the fast axis, from −10 to 10 V) (Figure 2a; inset) is compared with another resistance map obtained using the same voltage ranges for both gates but with the bottom gate sweep direction reversed (Figure 2b; inset). A direct comparison of these two resistance maps, especially in the regions enclosed by the cyan and orange dotted boxes, reveals a striking contrast in the resistance values of the insulating states.

To examine this behavior more closely, we acquired high‐resolution maps within the regions highlighted by the cyan dotted box in Figure 2b for both upward and downward sweeps. The resulting maps, replotted as a function of *n* and *D* in Figure 2c, reveal a significant discrepancy in resistance between the upward and downward scans within the insulating states near 𝜈 = −4. Notably, this insulating state is theoretically expected to host a substantial energy gap—on the order of tens of meV—regardless of the applied *D* field (Section S3), which would typically result in a resistance significantly larger than the resistance quantum, *h*/*e* ^2^ [37, 38]. Nevertheless, the upward sweep in the left panel of Figure 2c surprisingly reveals a pronounced reduction of resistance in the *D* > 0 V nm^−1^ region in contrast to the same region in the downward sweep shown on the right. We interpret this low‐resistance feature as a signature of anomalous metallic transport, considering the presence of a band gap at ν = −4 for the commensurate domains. The metallic behavior is further corroborated by the observed temperature dependence of resistance, which decreases as the temperature is lowered (Figure S3).

A qualitatively similar resistance response is also observed near the CNP, corresponding to the region enclosed by the orange dotted box in Figure 2b. This region is examined in greater detail in Figure 2d through a series of high‐resolution measurements with varying gate‐sweep ranges and directions. Each sweep reveals a small region of high resistance values—referred to here as a “resistive packet”—whose position shifts depending on gate sweep conditions. Based on the calculation in Figure 1b, the domains have an energy gap at the CNP in most of the *D* field values (except at discrete band inversion points); if the entire device were of a single domain, the resistance value at the CNP would exhibit insulating behavior. Unexpectedly, however, the resistance at the CNP is fairly low except in the case that the *D* field is tuned near the value of the resistive packet. The follow‐up temperature dependence measurements confirm that the system is insulating only when it is tuned to be at the resistive packet (Figure 2e).

This unusual transport response cannot be attributed solely to the electron transport in the commensurate domains of the hTG, which supposedly have band gaps at ν = −4 and 0 (CNP). Instead, the data point to the existence of an additional conductive channel that operates effectively in parallel to the conduction through the domains, thereby reducing the overall resistance, as illustrated schematically in the lower panel of Figure 2f. This parallel channel appears to exhibit a hysteretic response to the gate voltage sweep. At ν = −4 or 0, while the commensurate domains are nearly insulating with band gaps, as the phenomenology of *R*_xx_ map in Figure 1c strongly implies, the parallel conducting channel can lower the device resistance, effectively masking the insulating response of the domains. On the other hand, when this parallel channel becomes highly insulating, the total device resistance is close to that of the domains; i.e., that expected from the calculated band structure.

If this interpretation is correct, one should be able to isolate (extract) the ‘true’ conduction of the domains from the hysteretic parallel conduction by tuning the condition so that the parallel conduction becomes highly resistive. Figure 2g presents the device resistances at the CNP, extracted as line cuts from the dual‐gate maps in Figure 2d, where each panel corresponds to a different resistive packet position. Our analysis shows that the parallel path indeed becomes highly resistive near the resistive packet; consequently, the total resistance in that regime approaches and effectively reflects the intrinsic domain resistance (Section S4). By compiling an extensive set of additional resistance data obtained by varying the packet's positions (detailed protocols are described in the Experimental Section), we identify a clear trend. In the left panel of Figure 2h, it becomes apparent that the resistance values are bounded by a *D*‐field‐dependent ‘envelope’ profile, which we attribute to the inherent resistance contribution of the domains. Notably, this envelope is well explained by the calculated evolution of the band gap at the CNP—especially its closing and reopening as a function of the *D* field, as shown in the right panel of Figure 2h—thereby supporting our interpretation.

As a result, we can separately extract the resistance of the domains at the CNP and that of the parallel conduction, as shown in Figure 2i (see also Section S4). The left panel shows the domain resistance, while the right panel presents the resistances of the parallel conduction along the domain wall. The latter is plotted as two representative traces in black and purple, corresponding to the upward and downward sweeps, respectively. The profiles exhibit a local maximum at the packet position and remain low elsewhere, yielding a Gaussian‐like dependence on the *D* field.

It is worth mentioning that the hysteresis is consistently reproduced across multiple high‐quality hTG devices (Section S2), making it unlikely that the shorting path arises from extrinsic artifacts. Instead, given the known relaxation pattern of hTG, it is natural to conclude that the domain boundaries in relaxed hTG are the origin of the hysteretic parallel conduction path. As depicted in Figure 2f, the domain boundaries naturally form an interconnected network of conductive channels that bypass the gapped domains, effectively acting as a parallel pathway for transport. Notably, no comparable hysteresis is observed in our other moiré graphene systems, fabricated using the same process but lacking such domain boundaries—such as alternating twisted trilayer graphene (aTG), where the stacking configuration precludes their formation (Section S2). Together, the structural features of our system and experimental observations provide compelling evidence that the anomalous conduction originates from the domain boundaries.

### 2.3 Hall Response Accompanying Hysteretic Transport in hTG

**FIGURE 3:**
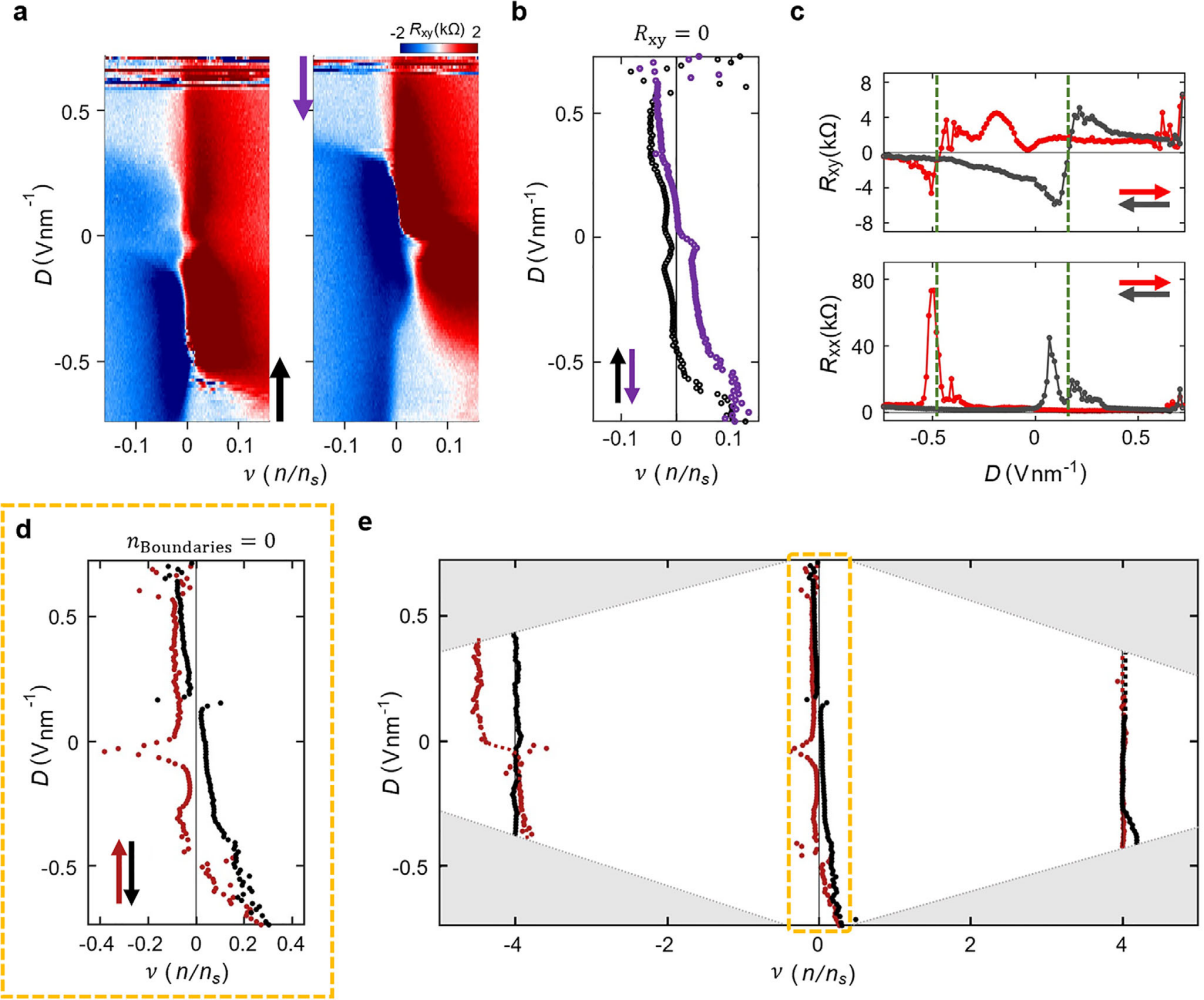
Hall measurements accompanying hysteretic transport in hTG. (a) Antisymmetrized Hall resistance, *R*_xy_ = (*R*_xy_(*B*_+_)‐*R*_xy_(*B*_‐_))/2, as a function of ν and *D* measured at *B*_±_ = ±0.2 T and T = 4 K for the hTG1 device during upward (black) and downward (purple) sweeps. (b) *R*_xy_ = 0 trajectories extracted from panel (a) for the upward (black) and downward (purple) sweeps, highlighting a hysteretic shift between the two sweep directions. Both trajectories deviate from the vertical grey line, which marks the CNP of the domains. (c) Line cuts of *R*_xy_ (top) and *R*_xx_ (bottom) at the CNP for each sweep. The green dotted lines mark the positions where *R*_xy_ = 0, coinciding with peaks in *R*_xx_. (d) Estimated CNP positions of the domain boundaries during upward and downward sweeps, indicated by red and black dots. (e) Collective plots of the extracted CNP positions of the domain boundaries at ν = −4, 0, and 4, plotted as a function of *n* and *D*.

To further explore the nature of the hysteretic signal, we performed Hall measurements, which offer useful insight into the charge carrier density and its variation with gate sweep conditions. Figure 3a displays the antisymmetrized Hall resistance maps acquired near the CNP for both upward and downward gate voltage sweeps. As in the case of the longitudinal resistance data, the Hall resistance exhibits clear hysteresis, and it is more clearly highlighted in Figure 3b, which shows the loci of points where the measured Hall resistance vanishes (*R*_xy_ = 0) for each sweep. Not only do the *R*_xy_ = 0 contours differ apparently between the two sweeps, but they also deviate from the ν = 0 line except for a couple of intersections. In contrast, the resistive packets consistently appear along the ν = 0 line, regardless of the sweep conditions (Section S4).

We also examined line‐cuts of both the Hall (*R*_xy_) and longitudinal (*R*_xx_) resistances for each sweep along the ν = 0 trajectory, as shown in the top and bottom panels of Figure 3c. Notably, *R*_xy_ vanishes only at the position where *R*_xx_ reaches a maximum—the location of the resistive packet—while being non‐zero at other points along the trajectory. Since the non‐zero value of *R*_xy_ implies the presence of net mobile carriers, these striking observations suggest that a finite transport‐active carrier contribution persists along the ν = 0 line, except for the *D* values where the resistive packet appears. Within our interpretation, the domains are close to charge neutral along the ν = 0 line, as supported by the fact that the main transport features attributed to the domains remain nearly fixed in carrier density irrespective of the hysteresis; the residual transport‐active carriers are thus attributed to the domain‐boundary network. We therefore conclude that the resistive packet marks the special condition where both the domains and the domain boundaries are charge neutral, whereas in surrounding regions at the CNP, the domains remain charge neutral but their boundaries retain a finite mobile‐carrier contribution.

The local carrier density of the domain boundaries (*n*_Boundaries_) can be estimated from the measured Hall resistance. Because the domains are insulating along the ν = 0 trajectory, electrical conduction is largely confined to the boundaries, making the measured Hall response a direct probe of their carrier content. Indeed, finite‐element simulations confirm that the carrier density scales approximately inversely with the Hall resistance, following *n* = *B*/(*eR*_xy_), regardless of the macroscopic network geometry (Section S5). Using the simulation, we extracted the carrier density of the domain boundaries at ν = 0 for each *D* field value and identified the gate voltage conditions at which they become charge neutral (Section S4). The estimated neutrality points, obtained from upward and downward sweeps, are plotted in Figure 3d using red and black markers, respectively. These neutrality trajectories trace out a closed loop that evolves with the applied *D* field. We further carried out analogous measurements near ν = ±4, and the results for all three filling factors are summarized in Figure 3e (Section S4). While the shapes of these trajectories differ between ν = −4, 0 and 4, they all exhibit hysteresis. Taken together, these results indicate that the sweep‐induced evolution of the charge neutrality condition of the domain boundaries underlies the anomalous transport behaviors observed in hTG.

## 3 Discussion

A key question emerges from our observation of gate‐sweep‐dependent charge filling: What is the origin of this behavior? In the absence of strain, hTG retains global inversion symmetry, and even after lattice relaxation, each domain preserves a local C_2y_ symmetry (or C_2x_, depending on the coordinate convention). This symmetry forbids a net out‐of‐plane polarization, making conventional ferroelectric‐switching pictures, such as charge redistribution [39, 40, 41] or relative interlayer sliding [42, 43, 44, 45], unlikely explanations for the present observations. Furthermore, such ferroelectric systems are generally not expected to involve substantial changes in the density of mobile carriers. Rather, they generate polarization through ion displacement or sliding‐induced structural rearrangement, which modifies the effective *D* field without directly changing the carrier density. By contrast, our measurements reveal a pronounced modulation, on the order of 10^11^ cm^−2^, in the number of mobile carriers participating in transport depending on the sweep history. These observations, therefore, point to a mechanism beyond simple ferroelectric switching.

**FIGURE 4:**
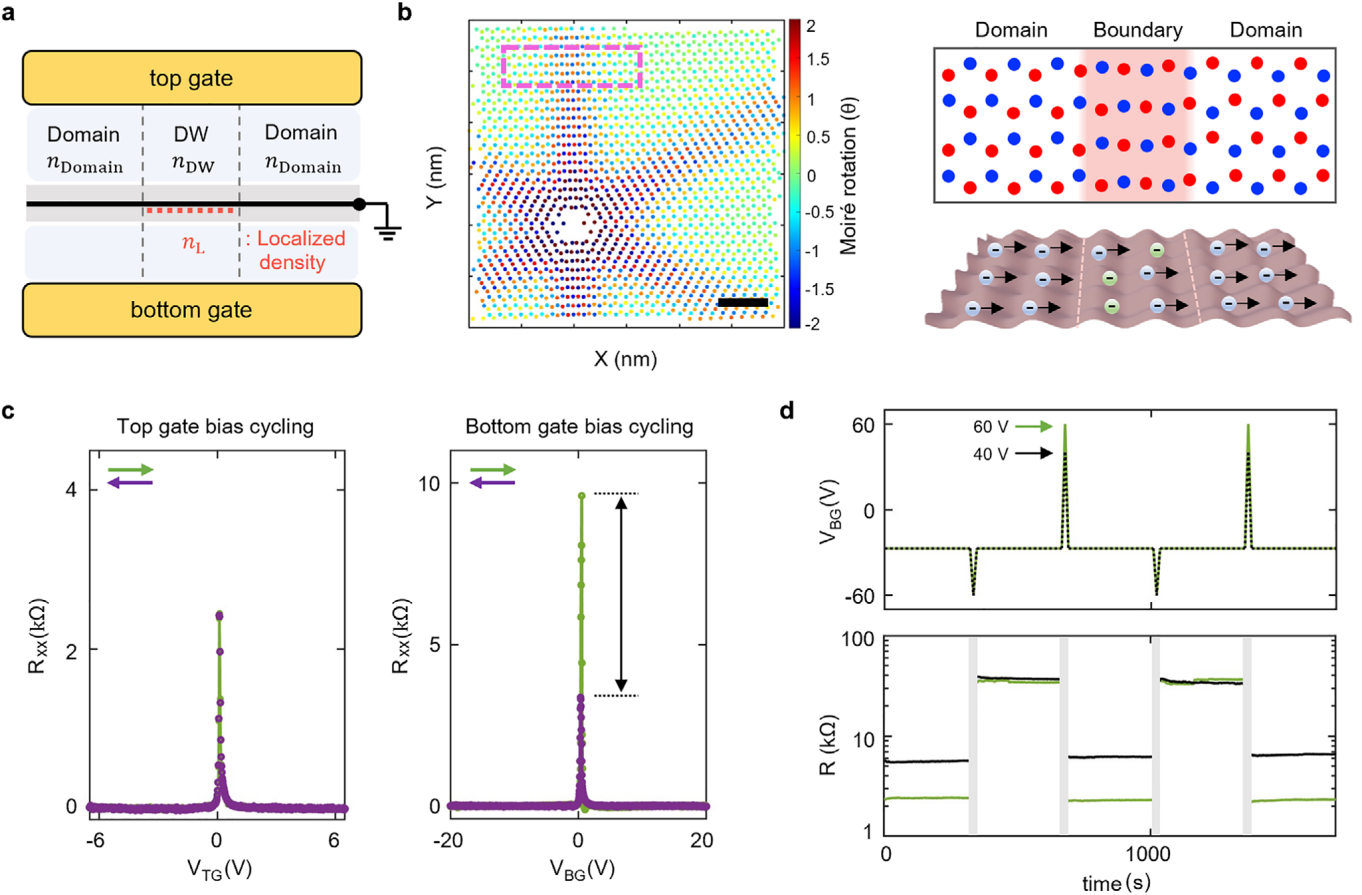
Electron localization as a possible origin of the anomalous transport of hTG. (a) Lattice simulation colored by local moiré rotation angle (scale bar represents 50 nm). (b) (Left) Schematic illustration of the moiré lattice together with its corresponding electron distribution. The domains host only mobile carriers, whereas both mobile and localized carriers coexist along the domain boundaries. (Right) Side‐view schematic illustrating the coexistence of localized states and mobile carriers along the domain boundaries. The charge density of localized states is denoted as *n*_L_, while the domains and boundaries host distinct charge densities, *n* and *n*_Boundaries_, which typically differ owing to the presence of the localized states. (c) Resistance measurements at 50 mK during cyclic sweeps of *V*_TG_ (left, from −10 to 10 V) and *V*_BG_ (right, from −60 to 60 V), with the opposite gate held fixed at 0 V. Hysteresis is observed only during *V*_BG_ sweeps, indicating a gate‐asymmetric response. (d) *R*_xx_ responses (bottom) to a series of *V*_BG_ excitations (top) with varying voltage pulse amplitudes. As examples, the black and green traces show the time evolution of *R*_xx_ in response to gate voltage excitation reaching 40 and 60 V, respectively, each driving the system into distinct stable resistance states.

Instead, we propose that this hysteretic charge density offset in *n*_Boundaries_ (see Figure 3d) is more naturally explained by the framework of electron localization [46, 47, 48, 49, 50, 51, 52, 53, 54, 55] arising from the deformation of the periodic moiré potential at the domain boundaries. Our lattice relaxation simulation (Figure 4b) of the twisting angle of 1.4° reveals that, even though the hTG is stacked to have globally‐twisted two rigid moiré lattices, the hTG readily relaxes to form periodic commensurate order in the domains while leaving the effect of twisting to be concentrated in the areas between the domains—i.e., domain boundaries—with strongly distorted moiré lattices. This structural environment provides a basis for a scenario, as suggested by previous theoretical studies [56, 57, 58, 59, 60, 61], where moiré‐incommensurability induces partial electron localization. Although an ideal straight domain wall may support dispersive modes along its longitudinal direction, the actual domain‐boundary structure in our device forms a triangular network with reduced dimensionality on the supermoiré scale, which may render the boundary states more susceptible to localization. In this picture, a portion of the electronic states at the aperiodic domain boundaries could be localized, while the electronic states within the periodic domains remain delocalized. Viewing our results through this framework suggests a consistent picture in which the hysteretic charge density originates primarily from these boundary regions.

Adopting this localization model allows us to contextualize our observations alongside recent experimental reports [50, 53, 54], which have attributed hysteretic responses to localized states in the presence of moiré potentials. Within this scenario, localized states are understood to reside outside the primary conduction channel; however, their occupation (represented as *n*_L_ in Figure 4b) can indirectly influence transport by altering the local electrostatic environment. A distinguishing feature of our observation, when interpreted this way, is that the effect of localized states appears spatially confined to the moiré‐incommensurate domain boundaries. Such aperiodic regions can be envisioned as hosting a coexistence of mobile carriers and localized electrons, with the latter remaining effectively decoupled from direct transport, as depicted in Figure 4b. Phenomenologically similar hysteretic behavior has also been reported to be sensitive to two moiré interfaces [55], which can be understood within the same context as our proposed model. Importantly, the mere presence of localized states would not by itself account for the hysteresis. Rather, the sweep‐direction‐dependent response may require Coulomb‐mediated coupling between localized and delocalized carriers, broadly analogous to interaction‐driven hysteretic scenarios discussed in earlier studies [50, 54].

Collectively, the observed behavior points toward a spatially non‐uniform carrier density between the domains and their boundaries under uniform gating. In our interpretation, the hysteretic charge filling is primarily associated with the domain boundaries rather than being distributed uniformly across the device. Although the resulting hysteresis is phenomenologically similar to ferroelectric (or ferroelectric‐like) behavior reported in several graphene‐based van der Waals heterostructures [50, 53, 54], the key distinction in our case lies in its spatially confined character. Namely, within our interpretation, the hysteretic behavior is spatially associated with the domain‐boundary network rather than the bulk domains. This spatially inhomogeneous response makes it less likely that the hysteresis originates solely from broadly distributed mechanisms, such as hBN‐related dielectric effects [46, 47, 51, 62] or graphene–hBN alignment‐induced moiré effects [50, 53, 54], although their contribution cannot be fully excluded. Instead, it is more naturally consistent with a localized‐state picture arising intrinsically from the moiré‐of‐moiré lattice structure (see also Section S6). Two additional features further support this interpretation: the pronounced gate asymmetry in the hysteretic response (Figure 4c and Figures S4 and S5) and the stable multiple resistance states (Figure 4d), both of which have previously been reported in systems hosting localized states [50, 51, 52, 53, 54, 55].

Despite these findings, several questions remain. In an ideal hTG domain, the local C_2y_ symmetry (or C_2x_, depending on the coordinate convention) would suggest a response symmetric with respect to ±*D*. However, the hysteresis loops in Figure 3e clearly violate this expectation and, moreover, as shown in Figure 4c, the anomalous response is dominant only for one gate‐sweep direction. In addition, a similar hysteretic response has not been reported in earlier hTG studies [33, 35]. One possible explanation is that the reconstructed domain‐boundary network in hTG is highly sensitive to the twist‐angle configuration and other structural details. For example, a finite twist‐angle difference between *θ*_12_ and *θ*_23_ could make the local boundary environments asymmetric, while the average twist angle may also influence the extent of lattice relaxation and the resulting boundary morphology. Out‐of‐plane corrugation or local strain could further distort the boundary structure. Such structural variations may influence the observed ±*D* asymmetry and, at the same time, modify the degree of local incommensurability at the boundaries, thereby affecting how strongly the hysteretic response develops. A more systematic study across devices with controlled twist‐angle difference, average twist angle, and structural morphology may therefore provide important clues for resolving these open questions in future work.

Finally, the robust and reproducible multistability observed in our hTG devices suggests potential for practical applications. These characteristics could be harnessed for multi‐level non‐volatile memory or neuromorphic computing components (Figure S6). Given the tunability afforded by twist angle, strain, and layer stacking in van der Waals heterostructures, our results underscore the potential of engineered aperiodic systems as a versatile platform for functional quantum materials in next‐generation electronic technology.

## 4 Experimental Section

### 4.1 Device Fabrication

Helical trilayer graphene devices were fabricated by sequentially stacking graphene flakes encapsulated in hexagonal boron nitride (hBN). The process began with the mechanical exfoliation of pristine hBN (30–50 nm thick) and monolayer graphene onto a SiO_2_/Si substrate (300 nm SiO_2_). Candidate flakes were identified via optical contrast under an optical microscope. To minimize strain and wrinkles, we adopted a ‘cut‐and‐stack’ approach [63], in which graphene flakes were pre‐cut using an atomic force microscope [64] rather than torn with hBN. The resulting pieces were assembled using a standard dry transfer method with a PDMS/polycarbonate (PC) stamp. The fully stacked heterostructures were then placed on a Si/SiO_2_ substrate, ensuring that all stacking and drop‐down steps were performed below 180°C to avoid unintentional interlayer rotation. No thermal annealing was applied after stacking, in order to preserve the intended twist angles. A Ti/Au top gate was deposited on the heterostructure, and the Hall bar geometry was defined by electron‐beam lithography followed by reactive ion etching. One‐dimensional edge contacts were formed by depositing Cr/Au electrodes using electron‐beam evaporation [65].

### 4.2 Simulation of Lattice Configuration

Structural relaxation was performed using the Large‐scale Atomic/Molecular Massively Parallel Simulator (LAMMPS) [66], an open‐source package for real‐space molecular modeling of systems with a large number of particles. Simulations were conducted on sufficiently large supercells to minimize edge effects and faithfully accommodate the unit cell geometry. The system was constructed by arranging three identical monolayer graphene sheets with a lattice constant of *a* = 0.246 nm and a nearest‐neighbor distance of *a*_0_ = 0.142 nm. The top two layers were vertically shifted to form an ABA‐stacked trilayer structure with an interlayer spacing of *c*_0_ = 0.335 nm. Identical twist angles were then applied between layers 1 and 2 and between layers 2 and 3. Intralayer interactions were modelled using the Brenner potential [67], while interlayer interactions were described by the Kolmogorov–Crespi potential [68]. Following relaxation, AA‐type stacking regions were identified between layers 1 and 2 (AA_12_, marked by red dots) and between layer 2and 3 (AA_23_, blue dots). The resulting domain and domain boundary patterns are qualitatively consistent with previous studies [30, 32].

### 4.3 LPFM Measurements

Lateral piezoresponse force microscopy (LPFM) measurements were carried out using a Cypher atomic force microscope (Asylum Research, Oxford Instruments). To directly image domain structures with high spatial resolution, LPFM characterization was performed at an intermediate stage of device fabrication, prior to final stacking. At this stage, the heterostructure was partially assembled by sequentially picking up the top hBN and the helical trilayer graphene (hTG) onto a PDMS/PC stamp supported by a glass slide. The stamp was subsequently inverted, exposing the hTG surface for direct LPFM characterization. Measurements were performed using PR‐E75 probes operated at their lateral contact resonance at approximately 0.9 MHz.

### 4.4 Transport Measurements

Electrical transport measurements were performed in a dilution refrigerator with a base temperature of 20 mK. A standard four‐probe measurement was employed, using lock‐in detection with an AC excitation current of 1 nA at 17.777 Hz. Voltage signals were recorded using SR865A and SR830 lock‐in amplifiers. The carrier density and displacement field were independently controlled by applying DC voltages to the Ti/Au top gate and the doped Si substrate, which served as a global bottom gate. Gate voltages were supplied using a GS200 voltage source. The charge carrier density, *n*, and displacement field, *D*, were determined using the following relations:

$$n=\left(c_{\mathrm{TG}}V_{\mathrm{TG}}+c_{\mathrm{BG}}V_{\mathrm{BG}}\right)/e,\;D=\left(c_{\mathrm{BG}}V_{\mathrm{BG}}-\;c_{\mathrm{TG}}V_{\mathrm{TG}}\right)/2\varepsilon_{0}$$

where *V*_TG_ and *V*_BG_ denote the voltages applied to the top and bottom gates, respectively; *ε*_0_ is the vacuum permittivity; *c* is the capacitance per unit area; and *e* is the elementary charge.

### 4.5 Gate‐Sweep Protocols for Mapping Resistance Envelopes

To compile the resistance envelope presented in Figure 2h, we performed 24 dual‐gate sweeps by systematically varying the *V*_BG_ sweep windows. Starting from a full‐range sweep of [−60 V, 60 V], the sweep boundaries were incrementally modified either by fixing the starting voltage at −60 V and reducing the ending voltage from 60 to 0 V in 12 V steps (e.g., [−60 V, 60 V] → [−60 V, 48 V] → ∙∙∙ → [−60 V, 0 V]), or by fixing the ending voltage at 60 V and increasing the starting voltage from −60 to 0 V in 12 V steps (e.g., [−60 V, 60 V] → [−48 V, 60 V] → ∙∙∙ → [0 V, 60 V]). The resistance envelope was constructed by aggregating data from both upward and downward *V*_BG_ scans across all measured ranges. This protocol drove the translocation of resistive packets, providing dense sampling across the entire accessible *D‐*field range, enabling the extraction of the *D*‐field‐dependent resistance profile of the domains.

### 4.6 Twist Angle Determination

The twist angle *θ* of each hTG device was determined from the Landau fan diagram by analyzing the quantum Hall states that emerged in the system. The degeneracy of these states was identified through Hall measurements, enabling us to extract the superlattice carrier density *n*_s_ from the slope of the corresponding fan features. Since the twist angle *θ* and *n*_s_ are related via the following expression, *θ* can be obtained accordingly:

$$n_{\nu =\pm 4}=\pm 8\mathrm{si}n^{2}\theta /\sqrt{3}a^{2}$$

where the graphene lattice constant is *a* = 0.246 nm. The values of *θ* obtained through this procedure were further verified by examining their consistency with the Brown–Zak oscillation sequences.

### 4.7 Electronic Band Calculation

The band structure of hTG was simulated using the Bistritzer–MacDonald approach. For the helically twisted trilayer system, we consider three layers (labeled l  =  1, 2, 3). In this structure, the outer layers are rotated in opposite directions by an angle *θ* while the middle layer reconstructs (shrinks), resulting in identical moiré lattices for the two bilayers. In each layer, the electron in the p_z_ orbital at lattice site *i* and sublattice *s* is denoted by |*i*, *l*, *s*〉 (with spin neglected due to weak spin–orbit coupling), and its Bloch wave function in momentum space $\tilde{k}$ is written as $|\tilde{k},l,s\rangle$. In the multilayer system, the full Hamiltonian couples different momentum states in different layers because the overall translational symmetry is broken. The sublattice position in each layer is modified by rotation and displacement as $r_{ls}=R_{\theta_{l}}\;.(r_{s}+d_{l}$), with **d**_1,2_ =  (0, 0) and for layer 3, **d**_3_ chosen as either $a(\frac{\sqrt{3}}{2},0)$ or *a*(0, 1) for domain and domain wall configurations, respectively. Focusing on electrons near a specific valley **K**_*l*′_ (writing $\tilde{k}$ **= K**_*l*_ + **k**, $\tilde{k^{\prime }}$ **= K**_*l*′_ + **k**′), the intralayer Hamiltonian is expressed as


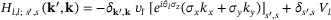

with Fermi velocity *v*_f_ = 10^6^ m/s and σ_*x*,*y*_ as Pauli matrices. The potential V_l_ models an external electric field with *V*_1_ =   − V, *V*_2_ =  0 and *V*_3_ =  V. The interlayer Hamiltonian is approximated by


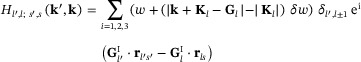

where to incorporate layer relaxation effect, the same‐sublattice hopping is reduced by a factor κ, modifying the term by [1 + (κ − 1)δ_s′,s _]. For our numerical calculations, we use the parameters *w*  =  110 meV, δ*w*  =   − 7 meV nm, and κ  =  0.7. A finite momentum mesh is generated connecting states through the interlayer coupling, and the full energy spectrum is obtained by diagonalizing the resultant Hamiltonian.

## Author Contributions

H.P., J.O., and J.J. conceived the project, H.P. and J.O. fabricated samples, performed measurements, and analyzed data. R.G., C.M., and B.‐J.Y. performed DOS calculations and contributed to the interpretation of data, J.J. performed the lattice relaxation simulations, K.W. and T.T. provided hBN crystals, B.‐J.Y. supervised the theoretical works, and J.J. supervised the overall project. All authors provided inputs and contributed to the writing of the manuscript. H.P. and J.O. contributed equally to the work.

## Conflicts of Interest

The authors declare no conflicts of interest.

## Supporting information




**Supporting File**: advs75919‐sup‐0001‐SuppMat.pdf.

## Data Availability

The data that support the findings of this study are available from the corresponding authors upon reasonable request.

## References

[1] N. W. Ashcroft and N. D. Mermin , Solid State Physics, (Holt, Rinehart and Winston, 1976).

[2] P. W. Anderson , “More Is Different,” Science 177 (1972): 393–396, 10.1126/science.177.4047.393. 17796623

[3] K. S. Novoselov , V. I. Fal'ko , L. Colombo , P. R. Gellert , M. G. Schwab , and K. Kim , “A Roadmap for Graphene,” Nature 490 (2012): 192–200, 10.1038/nature11458. 23060189

[4] A. K. Geim and I. V. Grigorieva , “Van der Waals Heterostructures,” Nature 499 (2013): 419–425, 10.1038/nature12385. 23887427

[5] Y. Liu , N. O. Weiss , X. Duan , H.‐C. Cheng , Y. Huang , and X. Duan , “Van der Waals Heterostructures and Devices,” Nature Reviews Materials 1 (2016): 16042, 10.1038/natrevmats.2016.42.

[6] R. Bistritzer and A. H. MacDonald , “Moiré Bands in Twisted Double‐Layer Graphene,” Proceedings of the National Academy of Sciences 108 (2011): 12233–12237, 10.1073/pnas.1108174108. PMC3145708 21730173

[7] Y. Cao , V. Fatemi , A. Demir , et al., “Correlated Insulator Behaviour at Half‐Filling in Magic‐Angle Graphene Superlattices,” Nature 556 (2018): 80–84, 10.1038/nature26154. 29512654

[8] G. Chen , L. Jiang , S. Wu , et al., “Evidence of a Gate‐tunable Mott Insulator in a Trilayer Graphene Moiré Superlattice,” Nature Physics 15 (2019): 237–241, 10.1038/s41567-018-0387-2.

[9] X. Lu , P. Stepanov , W. Yang , et al., “Superconductors, Orbital Magnets and Correlated States in Magic‐Angle Bilayer Graphene,” Nature 574 (2019): 653–657, 10.1038/s41586-019-1695-0. 31666722

[10] Y. Cao , V. Fatemi , S. Fang , et al., “Unconventional Superconductivity in Magic‐Angle Graphene Superlattices,” Nature 556 (2018): 43–50, 10.1038/nature26160. 29512651

[11] M. Yankowitz , S. Chen , H. Polshyn , et al., “Tuning Superconductivity in Twisted Bilayer Graphene,” Science 363 (2019): 1059–1064, 10.1126/science.aav1910. 30679385

[12] G. Chen , A. L. Sharpe , P. Gallagher , et al., “Signatures of Tunable Superconductivity in a Trilayer Graphene Moiré Superlattice,” Nature 572 (2019): 215–219, 10.1038/s41586-019-1393-y. 31316203

[13] J. M. Park , Y. Cao , K. Watanabe , T. Taniguchi , and P. Jarillo‐Herrero , “Tunable Strongly Coupled Superconductivity in Magic‐Angle Twisted Trilayer Graphene,” Nature 590 (2021): 249–255, 10.1038/s41586-021-03192-0. 33526935

[14] A. L. Sharpe , E. J. Fox , A. W. Barnard , et al., “Emergent Ferromagnetism Near Three‐Quarters Filling in Twisted Bilayer Graphene,” Science 365 (2019): 605–608, 10.1126/science.aaw3780. 31346139

[15] L. Wang , E.‐M. Shih , A. Ghiotto , et al., “Correlated Electronic Phases in Twisted Bilayer Transition Metal Dichalcogenides,” Nature Materials 19 (2020): 861–866, 10.1038/s41563-020-0708-6. 32572205

[16] M. Serlin , C. L. Tschirhart , H. Polshyn , et al., “Intrinsic Quantized Anomalous Hall Effect in a Moiré Heterostructure,” Science 367 (2020): 900–903, 10.1126/science.aay5533. 31857492

[17] G. Chen , A. L. Sharpe , E. J. Fox , et al., “Tunable Correlated Chern Insulator and Ferromagnetism in a Moiré Superlattice,” Nature 579 (2020): 56–61, 10.1038/s41586-020-2049-7. 32132694

[18] K. P. Nuckolls , M. Oh , D. Wong , et al., “Strongly Correlated Chern Insulators in Magic‐Angle Twisted Bilayer Graphene,” Nature 588 (2020): 610–615, 10.1038/s41586-020-3028-8. 33318688

[19] Y. Xie , A. T. Pierce , J. M. Park , et al., “Fractional Chern Insulators in Magic‐Angle Twisted Bilayer Graphene,” Nature 600 (2021): 439–443, 10.1038/s41586-021-04002-3. 34912084 PMC8674130

[20] R. Xiong , J. H. Nie , S. L. Brantly , et al., “Correlated Insulator of Excitons in WSe_2_/WS_2_ Moiré Superlattices,” Science 380 (2023): 860–864, 10.1126/science.add5574. 37167352

[21] Z. Zheng , Q. Ma , Z. Bi , et al., “Unconventional Ferroelectricity in Moiré Heterostructures,” Nature 588 (2020): 71–76, 10.1038/s41586-020-2970-9. 33230334

[22] M. Vizner Stern , Y. Waschitz , W. Cao , et al., “Interfacial Ferroelectricity by van der Waals Sliding,” Science 372 (2021): 1462–1466, 10.1126/science.abe8177. 34112727

[23] Y. Mao , D. Guerci , and C. Mora , “Supermoiré Low‐energy Effective Theory of Twisted Trilayer Graphene,” Physical Review B 107 (2023): 125423, 10.1103/PhysRevB.107.125423.

[24] J. Shi , J. Zhu , and A. H. MacDonald , “Moiré Commensurability and the Quantum Anomalous Hall Effect in Twisted Bilayer Graphene on Hexagonal Boron Nitride,” Physical Review B 103 (2021): 075122, 10.1103/PhysRevB.103.075122.

[25] X. Zhang , K.‐T. Tsai , Z. Zhu , et al., “Correlated Insulating States and Transport Signature of Superconductivity in Twisted Trilayer Graphene Superlattices,” Physical Review Letters 127 (2021): 166802, 10.1103/PhysRevLett.127.166802. 34723600

[26] Z. Zhu , S. Carr , D. Massatt , M. Luskin , and E. Kaxiras , “Twisted Trilayer Graphene: A Precisely Tunable Platform for Correlated Electrons,” Physical Review Letters 125 (2020): 116404, 10.1103/PhysRevLett.125.116404. 32975975

[27] X. Lin , C. Li , K. Su , and J. Ni , “Energetic Stability and Spatial Inhomogeneity in the Local Electronic Structure of Relaxed Twisted Trilayer Graphene,” Physical Review B 106 (2022): 075423, 10.1103/PhysRevB.106.075423.

[28] N. Nakatsuji , T. Kawakami , and M. Koshino , “Multiscale Lattice Relaxation in General Twisted Trilayer Graphenes,” Physical Review X 13 (2023): 041007.

[29] F. K. Popov and G. Tarnopolsky , “Magic Angle Butterfly in Twisted Trilayer Graphene,” Physical Review Research 5 (2023): 043079, 10.1103/PhysRevResearch.5.043079.

[30] J. Jang , “Effect of Lattice Relaxation on Electronic Spectra of Helically Twisted Trilayer Graphene: Large‐scale Atomistic Simulation Approach,” Journal of the Korean Physical Society 85 (2024): 727–736, 10.1007/s40042-024-01177-6.

[31] C. Yang , J. May‐Mann , Z. Zhu , and T. Devakul , “Multi‐moiré Trilayer Graphene: Lattice Relaxation, Electronic Structure, and Magic Angles,” Physical Review B 110 (2024): 115434, 10.1103/PhysRevB.110.115434.

[32] T. Devakul , P. J. Ledwith , L.‐Q. Xia , et al., “Magic‐angle Helical Trilayer Graphene,” Science Advances 9 (2023): adi6063, 10.1126/sciadv.adi6063. PMC10482339 37672575

[33] J. C. Hoke , Y. Li , Y. Hu , et al., “Imaging Supermoiré Relaxation in Helical Trilayer Graphene,” Nature Materials 25 (2026): 775–781, 10.1038/s41563-025-02423-3. 41492011

[34] Y. H. Kwan , P. J. Ledwith , C. F. B. Lo , and T. Devakul , “Strong‐Coupling Topological States and Phase Transitions in Helical Trilayer Graphene,” Physical Review B 109 (2024): 125141, 10.1103/PhysRevB.109.125141.

[35] L.‐Q. Xia , S. C. de la Barrera , A. Uri , et al., “Topological Bands and Correlated States in Helical Trilayer Graphene,” Nature Physics 21 (2025): 239–244, 10.1038/s41567-024-02731-6.

[36] D. Park , C. Park , K. Yananose , et al., “Unconventional Domain Tessellations in Moiré‐of‐Moiré Lattices,” Nature 641 (2025): 896–903, 10.1038/s41586-025-08932-0. 40369071

[37] M. P. Sarachik and S. V. Kravchenko , “Novel Phenomena in Dilute Electron Systems in Two Dimensions,” Proceedings of the National Academy of Sciences 96 (1999): 5900–5902, 10.1073/pnas.96.11.5900. PMC34203 10339515

[38] S. V. Kravchenko , D. Simonian , M. P. Sarachik , W. Mason , and J. E. Furneaux , “Electric Field Scaling at a B = 0 Metal‐Insulator Transition in Two Dimensions,” Physical Review Letters 77 (1996): 4938–4941, 10.1103/PhysRevLett.77.4938. 10062672

[39] K. Chang , J. Liu , H. Lin , et al., “Discovery of Robust in‐Plane Ferroelectricity in Atomic‐Thick SnTe,” Science 353 (2016): 274–278, 10.1126/science.aad8609. 27418506

[40] F. Liu , L. You , K. L. Seyler , et al., “Room‐Temperature Ferroelectricity in CuInP_2_S_6_ Ultrathin Flakes,” Nature Communications 7 (2016): 12357, 10.1038/ncomms12357. PMC4987531 27510418

[41] S. Yuan , X. Luo , H. L. Chan , et al., “Room‐Temperature Ferroelectricity in MoTe_2_ Down to the Atomic Monolayer Limit,” Nature Communications 10 (2019): 1775, 10.1038/s41467-019-09669-x. PMC6467908 30992431

[42] P. Meng , Y. Wu , R. Bian , et al., “Sliding Induced Multiple Polarization States in Two‐Dimensional Ferroelectrics,” Nature Communications 13 (2022): 7696, 10.1038/s41467-022-35339-6. PMC9744910 36509811

[43] W. Xue , P. Wang , W. Ci , et al., “Emergence of Sliding Ferroelectricity in Naturally Parallel‐stacked Multilayer ReSe_2_ Semiconductor,” Nature Communications 16 (2025): 6313, 10.1038/s41467-025-61756-4. PMC12238609 40628725

[44] A. Weston , E. G. Castanon , V. Enaldiev , et al., “Interfacial Ferroelectricity in Marginally Twisted 2D Semiconductors,” Nature Nanotechnology 17 (2022): 390–395, 10.1038/s41565-022-01072-w. PMC9018412 35210566

[45] X. Wang , K. Yasuda , Y. Zhang , et al., “Interfacial Ferroelectricity in Rhombohedral‐Stacked Bilayer Transition Metal Dichalcogenides,” Nature Nanotechnology 17 (2022): 367–371, 10.1038/s41565-021-01059-z. 35039684

[46] M. Onodera , K. Watanabe , M. Isayama , et al., “Carbon‐rich Domain in Hexagonal Boron Nitride: Carrier Mobility Degradation and Anomalous Bending of the Landau Fan Diagram in Adjacent Graphene,” Nano Letters 19 (2019): 7282–7286, 10.1021/acs.nanolett.9b02879. 31490080

[47] H. Wang , Y. Wu , C. Cong , J. Shang , and T. Yu , “Hysteresis of Electronic Transport in Graphene Transistors,” ACS Nano 4 (2010): 7221–7228, 10.1021/nn101950n. 21047068

[48] A. Sahoo , D. Nafday , T. Paul , et al., “Out‐of‐plane Interface Dipoles and Anti‐Hysteresis in Graphene‐Strontium Titanate Hybrid Transistor,” npj 2D Materials and Applications 2 (2018): 9, 10.1038/s41699-018-0055-5.

[49] R. M. Sterbentz , B. Kim , A. Flores‐Garibay , et al., “Gating Monolayer and Bilayer Graphene With a Two‐Dimensional Semiconductor,” npj 2D Materials and Applications 9 (2025): 29, 10.1038/s41699-025-00551-7.

[50] Z. Zheng , X. Wang , Z. Zhu , et al., “Electronic Ratchet Effect in a Moiré System: Signatures of Excitonic Ferroelectricity,” arXiv (2023), 10.48550/arXiv.2306.03922.

[51] D. Waters , D. Waleffe , E. Thompson , et al., “Anomalous Hysteresis in Graphite/Boron Nitride Transistors,” Nano Letters 25 (2025): 8768–8774, 10.1021/acs.nanolett.5c01799. 40376738

[52] T. F. Schranghamer , A. Oberoi , and S. Das , “Graphene Memristive Synapses for High Precision Neuromorphic Computing,” Nature Communications 11 (2020): 5474, 10.1038/s41467-020-19203-z. PMC7596564 33122647

[53] X. Yan , Z. Zhang , V. K. Sangwan , et al., “Moiré Synaptic Transistor With Room‐temperature Neuromorphic Functionality,” Nature 624 (2023): 551–556, 10.1038/s41586-023-06791-1. 38123805

[54] L. Zhang , J. Ding , H. Xiang , et al., “Electronic Ferroelectricity in Monolayer Graphene Moiré Superlattices,” Nature Communications 15 (2024): 10905, 10.1038/s41467-024-55281-z. PMC11685881 39738194

[55] G. Maffione , L. S. Farrar , and M. Kapfer , “Twist‐Angle‐Controlled Anomalous Gating in Bilayer Graphene/BN Heterostructures,” arXiv (2025), 10.48550/arXiv.2506.05548. 42410019

[56] T. Devakul and D. A. Huse , “Anderson Localization Transitions With and Without Random Potentials,” Physical Review B 96 (2017): 214201, 10.1103/PhysRevB.96.214201.

[57] M. Koshino , P. Moon , and Y.‐W. Son , “Incommensurate Double‐Walled Carbon Nanotubes as One‐Dimensional Moiré Crystals,” Physical Review B 91 (2015): 035405, 10.1103/PhysRevB.91.035405.

[58] M. J. Park , H. S. Kim , and S. Lee , “Emergent Localization in Dodecagonal Bilayer Quasicrystals,” Physical Review B 99 (2019): 245401, 10.1103/PhysRevB.99.245401.

[59] B. Huang and W. V. Liu , “Moiré Localization in Two‐dimensional Quasiperiodic Systems,” Physical Review B 100 (2019): 144202, 10.1103/PhysRevB.100.144202.

[60] A. Szabó and U. Schneider , “Mixed Spectra and Partially Extended States in a Two‐Dimensional Quasiperiodic Model,” Physical Review B 101 (2020): 014205.

[61] R. Ghadimi and B.‐J. Yang , “Quasiperiodic Pairing in Graphene Quasicrystals,” Nano Letters 25 (2025): 1808–1815, 10.1021/acs.nanolett.4c04386. 39869562

[62] K. Yasuda , X. Wang , K. Watanabe , T. Taniguchi , and P. Jarillo‐Herrero , “Stacking‐Engineered Ferroelectricity in Bilayer Boron Nitride,” Science 372 (2021): 1458–1462, 10.1126/science.abd3230. 34045323

[63] K. Kim , M. Yankowitz , B. Fallahazad , et al., “van Der Waals Heterostructures With High Accuracy Rotational Alignment,” Nano Letters 16 (2016): 1989–1995, 10.1021/acs.nanolett.5b05263. 26859527

[64] H. Li , Z. Ying , B. Lyu , et al., “Electrode‐Free Anodic Oxidation Nanolithography of Low‐Dimensional Materials,” Nano Letters 18 (2018): 8011–8015, 10.1021/acs.nanolett.8b04166. 30499679

[65] L. Wang , I. Meric , P. Y. Huang , et al., “One‐Dimensional Electrical Contact to a Two‐Dimensional Material,” Science 342 (2013): 614–617, 10.1126/science.1244358. 24179223

[66] A. P. Thompson , H. M. Aktulga , R. Berger , et al., “LAMMPS—A Flexible Simulation Tool for Particle‐based Materials Modeling at the Atomic, Meso, and Continuum Scales,” Computer Physics Communications 271 (2022): 108171, 10.1016/j.cpc.2021.108171.

[67] D. W. Brenner , O. A. Shenderova , J. A. Harrison , S. J. Stuart , B. Ni , and S. B. Sinnott , “A Second‐Generation Reactive Empirical Bond Order (REBO) Potential Energy Expression for Hydrocarbons,” Journal of Physics‐Condensed Matter 14 (2002): 783–802.

[68] A. N. Kolmogorov and V. H. Crespi , “Registry‐Dependent Interlayer Potential for Graphitic Systems,” Physical Review B 71 (2005): 235415, 10.1103/PhysRevB.71.235415.

