# Supplementary material for: Unconventional Hysteretic Charge Filling in Moiré‐Reconstructed Helical Trilayer Graphene
Source: Adv Sci (Weinh). 2026 Jun 1:e75919. Online ahead of print. doi: 10.1002/advs.75919 (PMC13336882; doi:10.1002/advs.75919)
Supplement: Supplementary file 1 — Supporting File: advs75919‐sup‐0001‐SuppMat.pdf. [file ADVS-9999-e75919-s001.pdf]

Supplementary Information for

Unconventional hysteretic charge filling in moiré-reconstructed helical trilayer graphene

Hangyeol Park†, Junhyeok Oh†, Rasoul Ghadimi, Chiranjit Mondal, Yungi Jeong, Won Beom Choi, Kenji Watanabe, Takashi Taniguchi, Bohm-Jung Yang* and Joonho Jang*

† These authors contributed equally to this work.

* correspondence to: joonho.jang@snu.ac.kr or bjyang@snu.ac.kr

Table of Contents

1. Additional data supporting the main figures

2. Supplementary analyses of individual samples

- **2.1. hTG1**
- **2.2. hTG2**
- **2.3. hTG3**
- **2.4. aTG1**

3. Electronic band structure calculations

- **3.1. Details of band structure calculations using the continuum model**
- **3.2. Band structures in domain and domain wall regions**
- **3.3. Band structure calculations of varying displacement fields**

4. Supplementary data on hysteretic transport behavior

- **4.1. Parallel resistance model interpretation of hysteresis**
- **4.2. Evolution of the resistive packet position in the *n*–*D* plane**
- **4.3. Hall resistance behavior at ±4 filling**

5. Finite-element analysis of Hall response

6. Discussion on the aperiodic moiré structure as an effective disorder

1. Additional data supporting the main figures

This section presents supporting data associated with the main figures in the main text. The figures included here offer additional experimental evidence and quantitative details relevant to the primary observations.

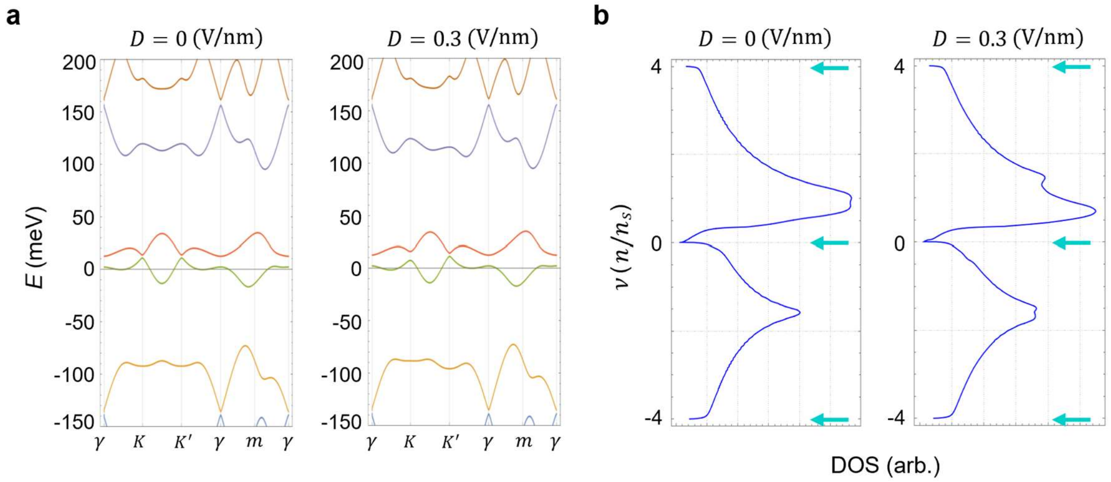

Fig. S1. Electronic band structure and density of states in relaxed domains.
(**a**) Electronic band structures calculated for relaxed domains at a twist angle of 1.35°, for a single K valley, shown at displacement fields of 0 V nm^{-1} (left) and 0.3 V nm^{-1} (right). The left panel is reproduced from the first panel of Fig. 1b in the main text. (**b**) Corresponding density of states (DOS) as a function of carrier density for displacement fields of 0 V nm^{-1} (left) and 0.3 V nm^{-1} (right). Cyan arrows mark the positions of energy gaps in each panel.

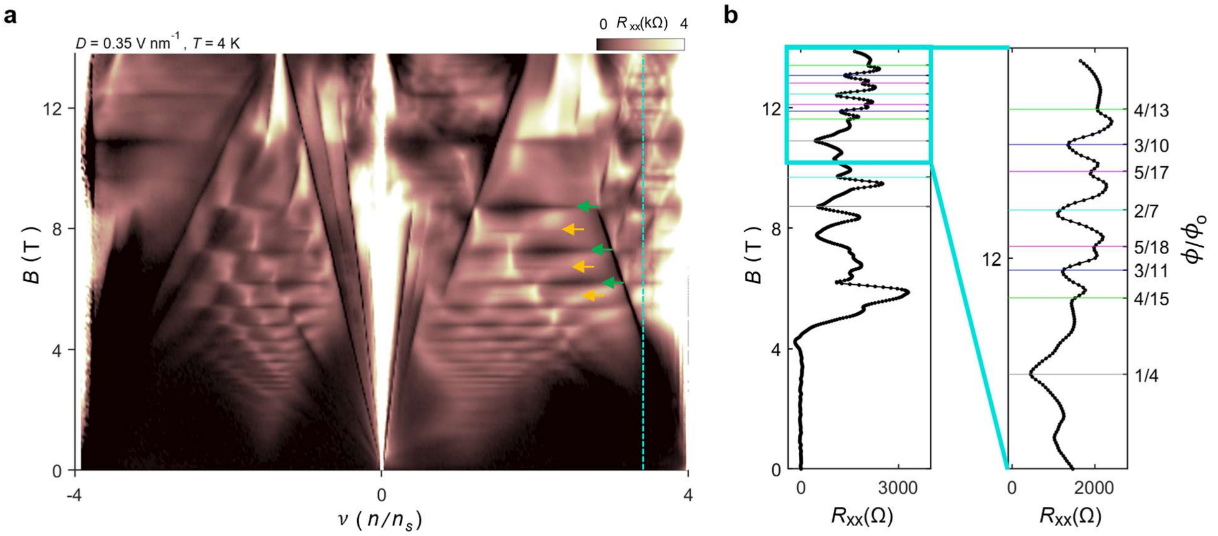

Fig. S2. High-order Brown–Zak (BZ) oscillations.
(**a**) Landau fan diagram at D = -0.35 V nm^{-1} and 4 K, reproduced from Fig. 1e. Green and orange arrows indicate the first- and second-order BZ oscillations, which appear as horizontal low-resistance features. At higher magnetic fields, particularly near the region marked by the cyan dashed line, higher-order oscillations up to fifth order are observed. (**b**) Line-cut of the resistance along the cyan dashed line in panel (a), plotted as a function of magnetic field. The full field range is shown on the left, with a magnified view of the region highlighted by the cyan box on the right. Each colored horizontal line marks the position of a BZ oscillation, labelled by its corresponding order.

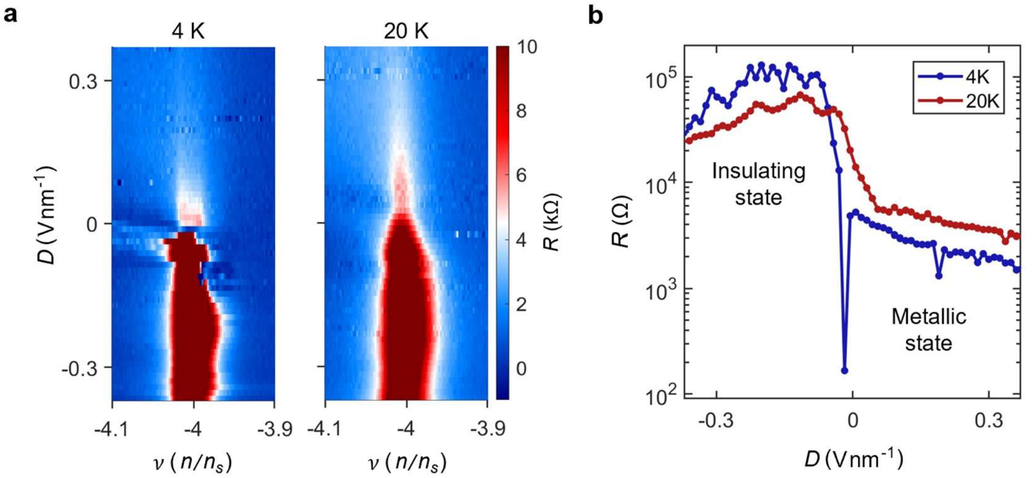

Fig. S3. Temperature-dependent resistance at ν = - 4.
(**a**) Longitudinal resistance map near ν = -4 as a function of n and D at 4 K (left) and 20 K (right). All data were acquired during upward gate voltage sweeps, as illustrated in the inset of Fig. 2B. (**b**) Maximum resistance values for each D from panel (a), plotted on a logarithmic scale. Red and blue points correspond to measurements at 4 K and 20 K, respectively. The region left of the grey dashed line exhibits insulating-like behavior, while the region to the right shows metallic-like behavior.

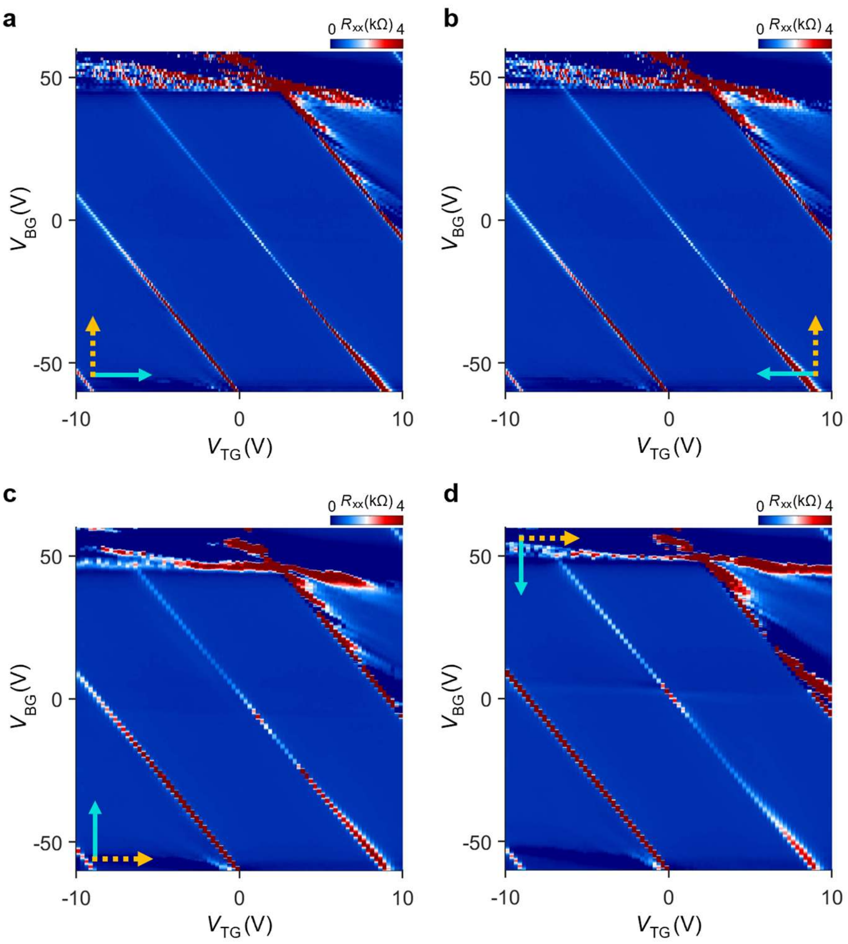

Fig. S4. Dual-gate sweep measurements with various sweep directions.
(**a**)–(**b**) Longitudinal resistance measured at 4 K, plotted as a function of bottom gate voltage (slow axis, swept upward in both panels) and top gate voltage (fast axis), swept forward in panel (a) and backward in panel (b). The two maps show minimal difference, indicating that the hysteresis is negligible when sweeping the top gate. (**c**)–(**d**) Same measurement as in panels (a) and (b), but with the top gate voltage as the slow axis (swept upward) and the bottom gate voltage as the fast axis, swept forward in panel (c) and backward in panel (d). In contrast to panels (a) and (b), a clear hysteretic difference emerges between panels (c) and (d).

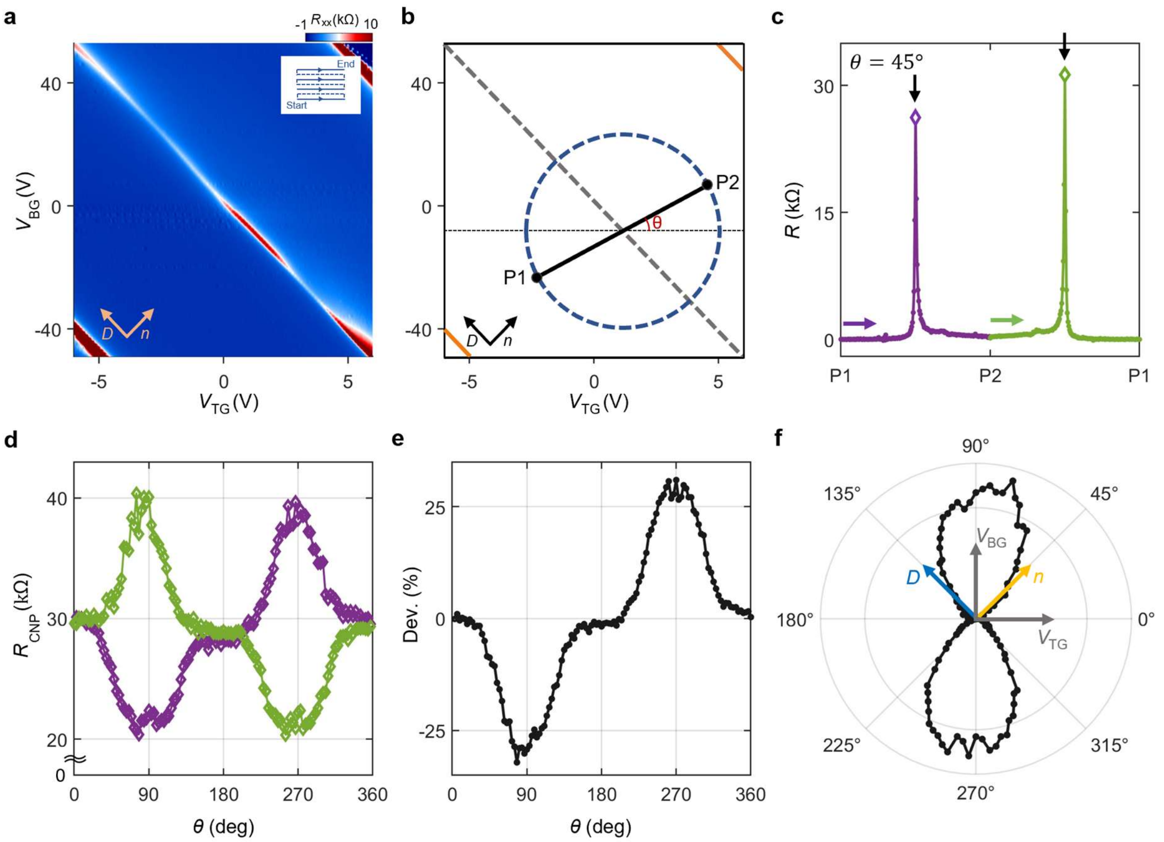

Fig. S5. Dependence of hysteresis on gate sweep angle.
(**a**) Four-probe longitudinal resistance measurement as a function of V_{TG} and V_{BG} for the hTG2 device with a twist angle of 1.66°. Arrows in the inset indicate the gate sweep directions used during the measurement. (**b**) Schematic highlighting the key features of panel (a). The grey dashed line and orange solid lines mark the CNP and band insulators ($\nu = \pm 4$), respectively. The blue dotted ellipse defines a set of points satisfying: $((V_{TG}\text{-}1.25)/V_{TG,0})^2+((V_{BG}\text{-}9)/V_{BG,0})^2 = 1$, where $V_{BG,0}$ = 3.8 V and $V_{BG,0}$ = 32.8 V. Two points, P1 and P2, are selected along the ellipse such that the line connecting them passes through its center and forms an angle θ with the horizontal axis. This angle defines the sweep trajectory used in other panels. (**c**) Longitudinal resistance measured for a representative sweep at θ = 45°, where gate voltages are swept from P1 to P2 (purple) and then from P2 to P1(green). (**d**) Maximum resistance values extracted from forward and backward sweeps as θ is varied from 0° to 360°, shown purple and green, respectively. (**e**)–(**f**) Relative resistance difference between the two sweeps in panel (c), defined as $\Delta R/R_{avg}$, plotted as a function of θ in Cartesian coordinates (e) and in polar coordinates (f). A gate-asymmetric hysteresis is observed between the two sweep directions.

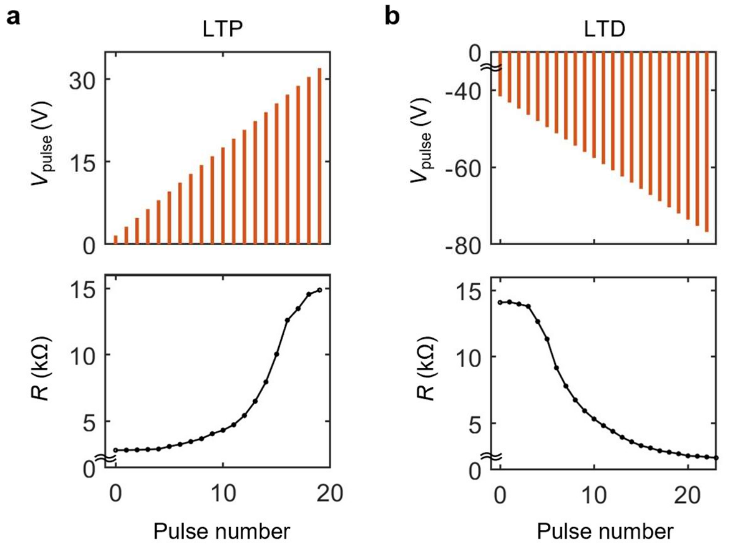

Fig. S6. Synaptic actions for neuromorphic computation.
(**a**)–(**b**) Multifunctional synaptic responses of long-term potentiation (LTP) and long-term depression (LTD) as a function of pulse number, measured on the hTG2 device with a twist angle of 1.66°. The initial state is set at (V_{TG}, V_{BG}) = (1.25 V, -9 V), a point on the CNP. From this state, additional pulse voltages are applied to V_{BG}. In both panels, the top subpanels show the applied pulse voltage (V_{Pulse}), and the bottom subpanels show the corresponding longitudinal resistance as a function of pulse number.

2. Supplementary analyses of individual samples

Among the over 40 hTG devices we fabricated with twist angles ranging from 1.3° to 1.8°, only three exhibited a uniform twist angle over several micrometers. Each of these samples showed the hysteretic behavior described in the main text, which was not observed in our alternating twisted trilayer device. The following are the measurement results for each sample.

2.1. hTG1

Device hTG1, the main device used in the main text, has a twist angle of 1.35° and exhibits the following characteristics.

(1) Uniform twist angle over a target area and robustness against thermal cycling

To verify whether the device maintains a uniform twist angle over a large area, we first performed two-probe resistance measurements between adjacent electrodes. **Fig. S7a** shows an OM image of the hTG1 device with electrode labels in red text. While sweeping the top gate, we measured the two-probe resistance between adjacent electrodes from electrode 1 to 8. The results are shown in **Fig. S7b**. In each measurement, three resistance peaks are observed, and their positions are identical. Considering that the spacing between peaks corresponds to the number of electrons required to fill a moiré band, this result indicates that each two-probe measurement region is defined by a single twist angle. Thus, the region above the red dashed line in **Fig. S7a** can be regarded as having a uniform twist angle.

Within this region, we performed four-probe measurements as functions of the dual gate using different configurations. In these two configurations, the current was applied from electrode 1 to 4, while the resistance was measured between electrodes 2 and 3 in one case (**Fig. S7c**) and between electrodes 6 and 7 in the other (**Fig. S7d**). In each measurement, the gate sweep was performed downward, as indicated in the insets of each figure. The two maps exhibit qualitatively similar features, further implying the uniformity of the sample. Notably, these measurements were taken during a different thermal cycle than the data presented in **Fig. 2a** of the main text, emphasizing that our results are also robust against thermal cycling. Both the data presented in the main text and the following figures were measured using the configuration shown in **Fig. S7d**.

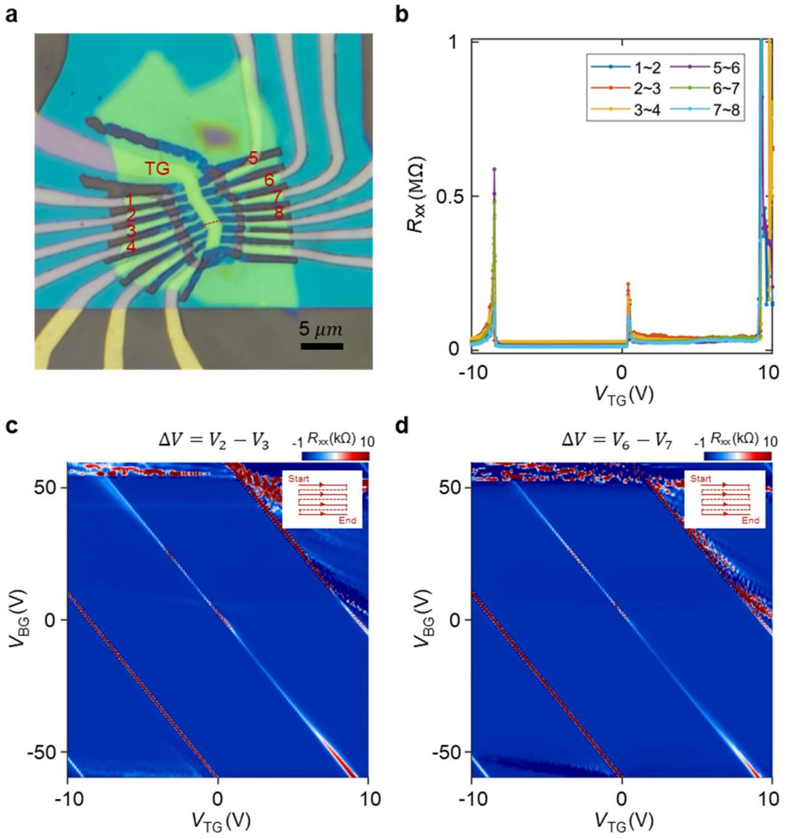

Fig. S7. Device characterization and additional transport measurements of hTG1.
(**a**) Optical image of the hTG1 device with a 5 μm scale bar. Electrode labels are shown in red text. (**b**) Two-probe resistance measured at 50 mK under different configurations. (**c**)–(**d**) Dual-gate sweep results obtained at 2.5 K using four-probe measurements between electrodes 2 and 3 (c), and between electrodes 3 and 4 (d). The current was applied from electrode 1 to 4. In both cases, the bottom gate was swept downward as the slow axis.

(2) Modification of the Hofstadter spectra by *D* field

In **Fig. 1c** of the main text, we explained that the two local resistance minima observed in the resistance plot as a function of the *D* field near the CNP correspond to topological band inversions predicted by band calculations. During these transitions, the Chern numbers of the two bands closest to the CNP change following the topological band inversion, which can modify the Hofstadter spectra. Considering the behavior of the Hofstadter gap at the CNP under varying magnetic fields, it is well established that in systems with trivial topology, such gaps remain open and do not close [1]. Therefore, the gap closing observed here can be attributed to the intersecting Hofstadter subbands of distinct flavors, including spin and valley, and is consequently influenced by the topology of the involved bands [2].

For further investigation, we performed magnetoresistance measurements at $D = 0$ V nm^{-1} and T = 1 K, with the results shown in **Fig. S8a**. Notably, the gap at the CNP (high resistive region near $\nu = 0$) closes around 8 T, as can be seen in the cyan dotted box. This finding stands in stark contrast to the results obtained in the presence

of a D field, as shown in **Fig. 1d**, where the gap at the corresponding position remains open. To examine the dependence of the Hofstadter gap at the CNP on the D field, we measured the resistance near the CNP while varying the D field under a magnetic field of 11.5 T (**Fig. S8b**). In this plot, the high resistance near the CNP sharply drops precisely at the positions of the local resistance minima in **Fig. 1c** (marked by pink arrows), which were predicted to correspond to topological transition points. This observation indirectly supports the presence of topological transitions at these positions.

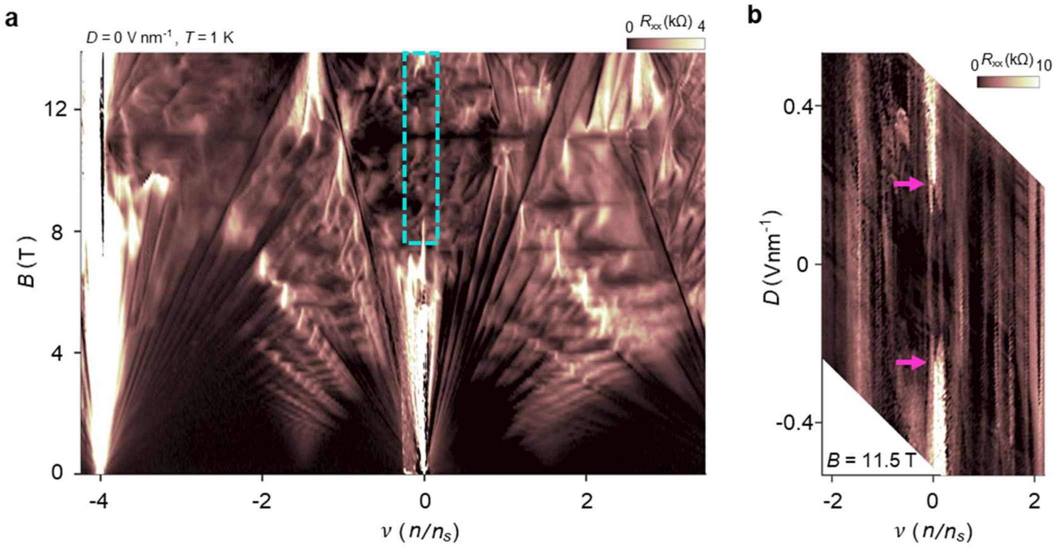

Fig. S8. Displacement field-dependent Hofstadter gap in hTG1.
(**a**) Landau fan diagrams of longitudinal resistance measured from the hTG1 at $D = 0$ V nm^{-1}and T = 1 K. The cyan dashed box highlights the region where the Hofstadter gap at the CNP closes. (**b**) Resistance map near the CNP as a function of the D field at 11.5 T. A sharp resistance drop is observed at the pink arrows, which mark the expected positions of topological band inversions.

2.2. hTG2

hTG2 is the device used in **Fig. S5** and **S6**, with a twist angle of 1.66°. Due to its larger twist angle compared to hTG1, access to the band insulators is more limited. Still, it exhibits qualitatively the same hysteretic behavior as hTG1, as detailed below.

(1) Basic characterization and hysteretic response

The hTG2 device has the same geometry as hTG1. **Fig. S9a** shows an OM image of the hTG2 device with electrode labels indicated in red. The four-probe measurement map in the n-D plane is shown in **Fig. S9b**, obtained by applying current from electrode 1 to 4 and measuring the resistance between electrodes 2 and 3. During this measurement, the bottom gate was swept in the upward direction. Similar to the hTG1 device, the map reveals high-resistance vertical lines corresponding to $\nu = 0$ and ± 4. Additionally, the resistance at the CNP exhibits two local minima, as indicated by the pink arrows, which are predicted to correspond to topological band inversions. To investigate the hysteretic behavior, we rescanned the resistance near the CNP in both the upward and downward

sweep directions, as shown in **Fig. S9c**. Consistent with the results in **Fig. 2** of the main text, hysteresis appears depending on the sweep direction, while the D field positions of the dips near the CNP remain unchanged, as indicated by the pink dotted lines.

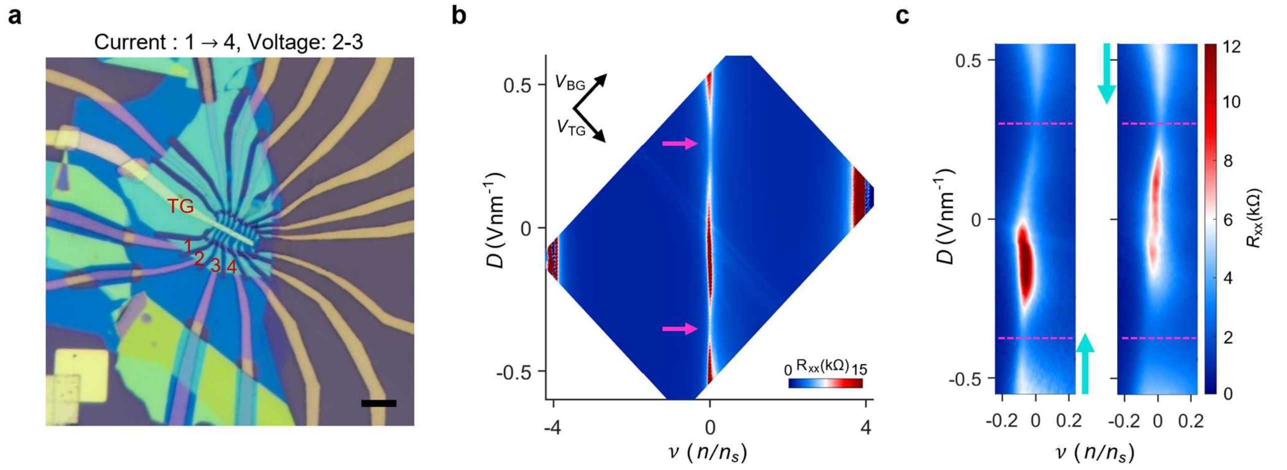

Fig. S9. Characterization and hysteretic transport in hTG2.
(**a**) Optical image of the hTG2 device with a 10 μm scale bar. Electrode labels are indicated in red. (**b**) Longitudinal resistance map of hTG2 as a function of ν and D at 50 mK. Resistance was measured between electrodes 2 and 3 while applying current from electrode 1 to 4. As in the hTG1 device, the resistance near the CNP exhibits two local minima as the D field is varied, marked by pink arrows. (**c**) Hysteresis of resistance near the CNP under opposite sweep directions of the D field. Measurements were performed at 4 K. A cyan arrow in each plot indicates the direction of the sweep.

(2) Magnetoresistance measurements at various D field

This device also exhibits modifications to the Hofstadter spectra as a function of the D field. Magnetoresistance measurements at 100 mK for D = 0 V nm^{-1} and D = -0.5 V nm^{-1} are shown in **Fig. S10a** and **b**, respectively. Similar to the case of the hTG1 device, the Hofstadter gap at the CNP closes when D = 0 V nm^{-1}, as indicated by the cyan dashed box. In contrast, when D is applied, the gap remains open up to 14 T, consistent with the behavior observed in hTG1. Notably, the Landau fans on the electron side appear less developed than on the hole side, as seen in **Fig. S10a**. This is likely due to the electron-side band becoming flatter as the twist angle of this sample approaches the magic angle of 1.8°.

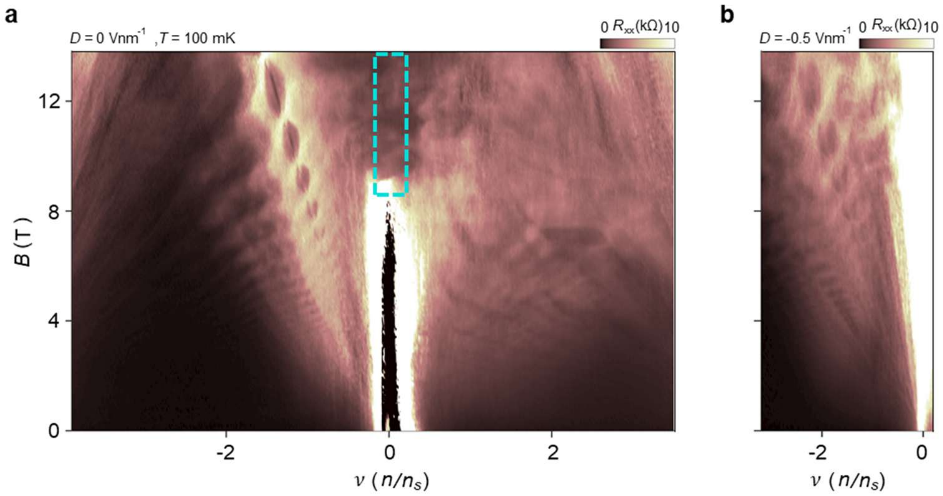

Fig. S10. Displacement field-tuned Hofstadter gap closure in hTG2.
(**a**)–(**b**) Landau fan diagrams of longitudinal resistance for hTG2 at D = 0 V nm^{-1} (a) and D = -0.5 V nm^{-1} (b) at T = 100 mK. When D = 0 V nm^{-1}, the Hofstadter gap is closed as indicated by the cyan dashed box, whereas at D = −0.5 V nm^{-1}, the corresponding region becomes insulating.

2.3. hTG3

We have another sample that exhibits hysteretic behavior, referred to as hTG3, with a twist angle of approximately 1.7° estimated from its geometric capacitance. The OM image of this device and the measurement configuration are shown in **Fig. S11a**. The dual-gate sweep results in **Fig. S11b** and **c** indicate that it qualitatively shares the same hysteretic behavior as other hTG devices. We confirmed that this sample also exhibits hysteresis exclusively in the bottom gate sweep.

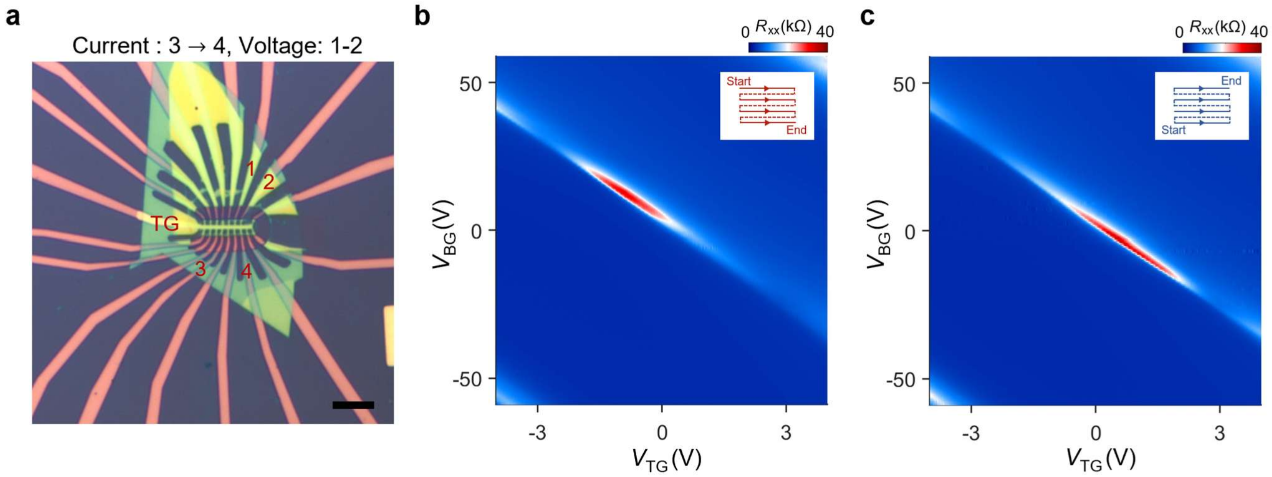

Fig. S11. Characterization and hysteretic transport in hTG3.
(**a**) Optical image of the hTG3 device with a 10 μm scale bar. Electrode labels are indicated in red. (**b**)–(**c**) Dual-gate resistance measurements at 1.8 K as functions of the bottom gate voltage (slow axis, swept upward in both panels) and the top gate voltage (fast axis), with V_{TG} swept downward in (b) and upward in (c). Resistance was measured between electrodes 1 and 2 while applying current from electrode 3 to 4. As in other hTG devices, the resistance near the CNP exhibits distinctive hysteretic behavior.

2.4. aTG1

To confirm that the hysteretic behavior observed in hTG devices originates from the system itself rather than being an artifact of the fabrication process, we fabricated an alternating twisted trilayer graphene device, referred to as aTG1, using the same fabrication process as the hTG devices. Unlike the hTG system, the alternating twisted trilayer graphene system has its two moiré patterns—one formed between the top and middle graphene layers and the other between the middle and bottom layers—perfectly aligned. As a result, commensurate moiré sites extend across the entire sample, preventing the formation of domain boundaries and the associated hysteresis.

Fig. S12a shows an OM image of the aTG1 device, which shares the same geometry as the hTG devices. Among the many electrodes in the device, those in cleaner regions were selected to configure the measurement setup: current was applied from electrode 1 to 4 while measuring the resistance between electrodes 2 and 3. First, we grounded the top gate and performed magnetotransport measurements at 50 mK by sweeping the back gate from -60 V to 60 V, using the same range used for the hTG devices, with the resulting data shown in **Fig. S12b**. The Landau fans are well developed at a few teslas, from which the twist angle was calculated to be 1.58°. We also performed measurements while varying the temperature from 50 mK to 2 K, as shown in **Fig. S12c**, where a superconducting dome with a T_c of approximately 700 mK was observed. These results indicate that the fabricated structure is a well-formed alternating twisted trilayer graphene system [3,4].

To examine whether hysteresis occurs, we set the temperature to 1 K, above the superconducting transition, and measured the resistance while sweeping the bottom gate forward and backward. The results, shown in **Fig.**

S12d, include green and purple traces, which correspond to the forward and backward sweeps, respectively. Importantly, no hysteresis is observed between the two sweeps, reinforcing that the distinct hysteretic behavior observed in the hTG system originates from the system itself.

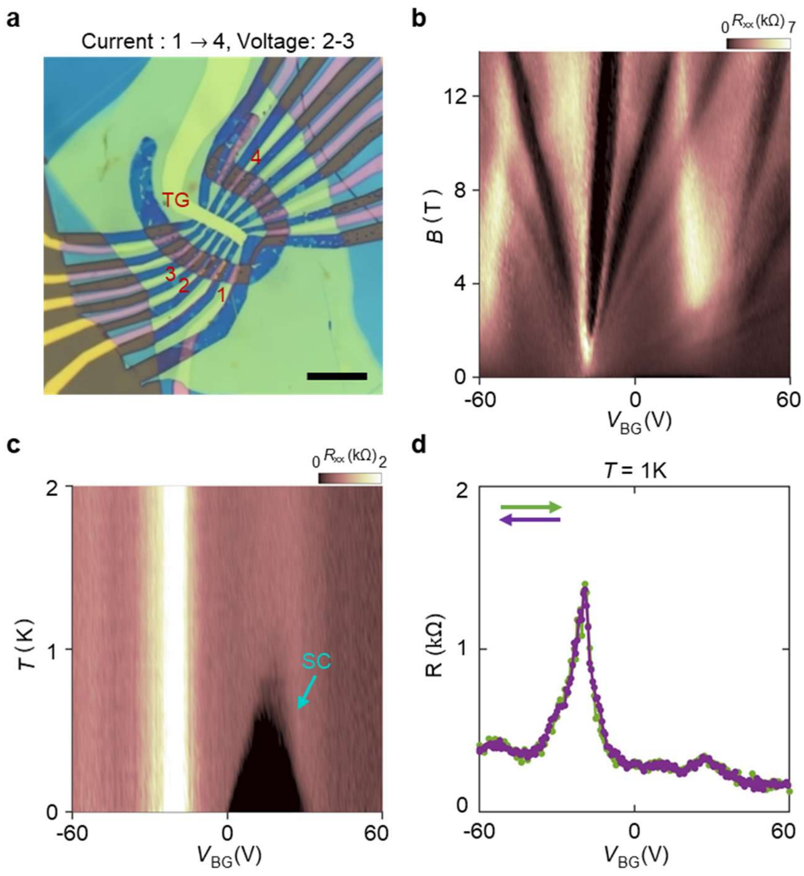

Fig. S12. Characterization and non-hysteretic transport in aTG1.
(**a**) Optical image of aTG1 device with a 10 μm scale bar. The red annotations in the image indicate the electrode labels. (**b**) Magnetoresistance measurement at 50 mK while sweeping the bottom gate from -60 V to 60 V, with the top gate held at 0 V. The twist angle extracted from the Landau fan is approximately 1.58°. (**c**) Temperature-dependent resistance map at zero magnetic field. A superconducting dome with T_c of approximately 700 mK exists in the bottom gate voltage range of 0 V to 30 V. (**d**) Line cut plot at 1 K extracted from the map in (c). The green and purple data correspond to measurements taken while increasing and decreasing the gate voltage, respectively, indicating the absence of hysteresis.

3. Electronic band structure calculations

In this section, we provide details regarding the electronic band structure calculations presented in the main text, along with supplementary data to aid further understanding.

3.1. Details of band structure calculations using the continuum model

The band structure of hTG was simulated using the Bistritzer–MacDonald approach. A single graphene layer is described by the real-space lattice vectors $\mathbf{a}_1 = a\,(\sqrt{3}, 0)$ and $\mathbf{a}_2 = a\,(\frac{\sqrt{3}}{2}, \frac{3}{2})$, and the corresponding reciprocal lattice vectors $\mathbf{b}_1 = \frac{2\pi}{a}\left(\frac{1}{\sqrt{3}}, -\frac{1}{3}\right)$ and $\mathbf{b}_2 = \frac{2\pi}{a}\left(0, \frac{2}{3}\right)$ with $a = 1.42 \times 10^{-10}$ m. The two sublattices are located at $\mathbf{r}_1 = a(0,0)$ and $\mathbf{r}_2 = a\,(0,1)$. At half filling, Dirac cones appear at the inequivalent valleys $\mathbf{K}_0 = \frac{4\pi}{3\sqrt{3}a}$ and $\mathbf{K}_0' = -\frac{4\pi}{3\sqrt{3}a}$, where the low-energy excitations near $\mathbf{K}_0$ are described by the Dirac Hamiltonian

$$H_0 = v_{\mathrm{f}}\,(\sigma_x k_x + \sigma_y k_y),$$

with Fermi velocity $v_f = 10^6$ m/s and $\sigma_{x,y}$ as Pauli matrices.

For the helically twisted trilayer system, we consider three layers (labelled $l = 1,2,3$). In this structure, the outer layers are rotated in opposite directions by an angle θ while the middle layer reconstructs (shrinks), resulting in identical moiré lattices for the two bilayers. In each layer, the electron in the p_z orbital at lattice site i and sublattice s is denoted by $|i, l, s\rangle$ (with spin neglected due to weak spin–orbit coupling). Taking advantage of the translational symmetry within each layer, we introduce the momentum-space basis

$$|\tilde{\mathbf{k}}, l, s\rangle = (1/\sqrt{\mathrm{N}}) \sum_i \mathrm{e}^{\mathrm{i}\,\tilde{\mathbf{k}}\cdot\mathbf{r}_l} |i, l, s\rangle,$$

where N is the number of lattice sites per layer. Assuming a tight-binding hopping $t(\mathbf{r}_J - \mathbf{r}_l)$ that depends on both distance and direction, the Hamiltonian in momentum space is

$$H_{l',l;s',s}(\tilde{\mathbf{k}}', \tilde{\mathbf{k}}) = 1/\mathrm{N} \sum_{i,j} \mathrm{e}^{\mathrm{i}\,(\tilde{\mathbf{k}}'\cdot\mathbf{r}_J - \tilde{\mathbf{k}}\cdot\mathbf{r}_l)} \mathrm{t}(\mathbf{r}_J - \mathbf{r}_l).$$

For an isolated layer, this Hamiltonian is block diagonal ($\tilde{\mathbf{k}}' = \tilde{\mathbf{k}}$) and yields Dirac cones centred at $\mathbf{K}_l$ and $\mathbf{K}_l'$. However, in the multilayer system, the full Hamiltonian couples states in different layers because the overall translational symmetry is broken. The sublattice position in each layer is modified by rotation and displacement as $\mathbf{r}_{ls} = R_{\theta_l}\cdot(\mathbf{r}_s + \mathbf{d}_l)$, with $\mathbf{d}_{1,2} = (0,0)$ and for layer 3, $\mathbf{d}_3$ chosen as either $a(\frac{\sqrt{3}}{2}, 0)$ or $a(0,1)$ for domain and domain wall configurations, respectively.

The interlayer hopping for the p_z orbitals is expressed in Fourier space as

$$t(\mathbf{r}) = \int d^2q\, t(|\mathbf{q}|)\, e^{-i\mathbf{q}\cdot\mathbf{r}},$$

and by writing the position as $\mathbf{r}_l = \mathbf{R}_l + \mathbf{r}_{ls}$, the momentum-space Hamiltonian becomes

$$H_{l',l;\,s',s}(\tilde{\mathbf{k}}', \tilde{\mathbf{k}}) = 1/N \int d^2q\, t(|\mathbf{q}|) \sum_j \sum_i e^{i(\tilde{\mathbf{k}}'\cdot\mathbf{R}_j + \tilde{\mathbf{k}}'\cdot\mathbf{r}_{l's'} - \tilde{\mathbf{k}}\cdot\mathbf{R}_i - \tilde{\mathbf{k}}\cdot\mathbf{r}_{ls})} e^{-i\mathbf{q}\cdot(\mathbf{R}_j + \mathbf{r}_{l's'} - \mathbf{R}_i - \mathbf{r}_{ls})}.$$

After summing over lattice sites, delta functions enforce momentum conservation modulo reciprocal lattice vectors. Focusing on electrons near a specific valley (writing $\tilde{\mathbf{k}} = \mathbf{K}_l + \mathbf{k}$, $\tilde{\mathbf{k}}' = \mathbf{K}_l' + \mathbf{k}'$), the interlayer coupling is written as

$$H_{l',l;\,s',s}(\tilde{\mathbf{k}}', \tilde{\mathbf{k}}) = 1/\Omega \sum_{\mathbf{G}_l, \mathbf{G}_{l'}} t(|\mathbf{k} + \mathbf{K}_l - \mathbf{G}_l|)\, \delta_{\mathbf{k}'-\mathbf{k}, \mathbf{K}_l - \mathbf{K}_{l'} + \mathbf{G}_{l'} - \mathbf{G}_l}\ e^{i(\mathbf{G}_{l'}\cdot\mathbf{r}_{l's'} - \mathbf{G}_l\cdot\mathbf{r}_{ls})}.$$

Since $t(|q|)$ decays rapidly with increasing q, only three sets of reciprocal lattice vectors (indexed by $i = 1,2,3$) contribute significantly. We approximate

$$t(|\mathbf{k} + \mathbf{K}_l - \mathbf{G}_l|)/\Omega = w + (|\mathbf{k} + \mathbf{K}_l - \mathbf{G}_l| - |\mathbf{K}_l|)\, \delta w,$$

with $w = t(|\mathbf{K}_l|)$ and $\delta w = \frac{dt(q)}{dq}|_{q=|\mathbf{K}_l|}$. Then, the interlayer Hamiltonian is then given by

$$H_{l',l;\,s',s}(\mathbf{k}', \mathbf{k}) = \sum_{i=1,2,3} (w + (|\mathbf{k} + \mathbf{K}_l - \mathbf{G}_l| - |\mathbf{K}_l|)\, \delta w)\ \ \delta_{l',l\pm1}\ \ e^{i(\mathbf{G}_{l'}^i\cdot\mathbf{r}_{l's'} - \mathbf{G}_l^i\cdot\mathbf{r}_{ls})}.$$

To incorporate layer relaxation effect, the same-sublattice hopping is reduced by a factor κ, modifying the term by $[1 + (\kappa - 1)\delta_{s',s}]$ (with $\kappa = 0$ defining the chiral limit).

The intralayer Hamiltonian is expressed as

$$H_{l,l;\,s',s}(\mathbf{k}', \mathbf{k}) = -\delta_{\mathbf{k}',\mathbf{k}}\, v_f\, [e^{i\,\theta_l \sigma_z}(\sigma_x k_x + \sigma_y k_y)]_{s',s} + \delta_{s',s} V_l,$$

where the potential V_l models an external electric field with $V_1 = -V$, $V_2 = 0$, and $V_3 = V$.

For our numerical calculations, we use the parameters $w = 110$ meV, $\delta w = -7$ (in suitable units), and $\kappa = 0.7$. A momentum mesh is generated connecting states through the interlayer coupling, and the full energy spectrum is obtained by diagonalizing the resultant Hamiltonian. This approach captures the low-energy physics of the twisted trilayer graphene system while retaining the essential details of the original tight-binding and Fourier analysis.

3.2. Band structures of domain and domain wall regions

When considering the relaxed lattice structure, the system consists of domains surrounded by aperiodic domain boundaries, as illustrated in the panels of **Fig. S13a**. As shown in previous references [5-7] and our simulations [8], the system contains two types of domains related by C_{2z} symmetry, commonly referred to as h-

domain and $\bar{h}$-domain (see right panel). Each domain is characterized by its own displacement vector, d_1 and d_2, which are respectively defined by the relative shift between moiré sites associated with different moiré patterns, as illustrated in the first and third panels of **Fig. S13b**. Since these domains exhibit well-defined periodicity, as indicated by the wavelength λ_m in the first panel, their band structure can be computed within the continuum approximation.

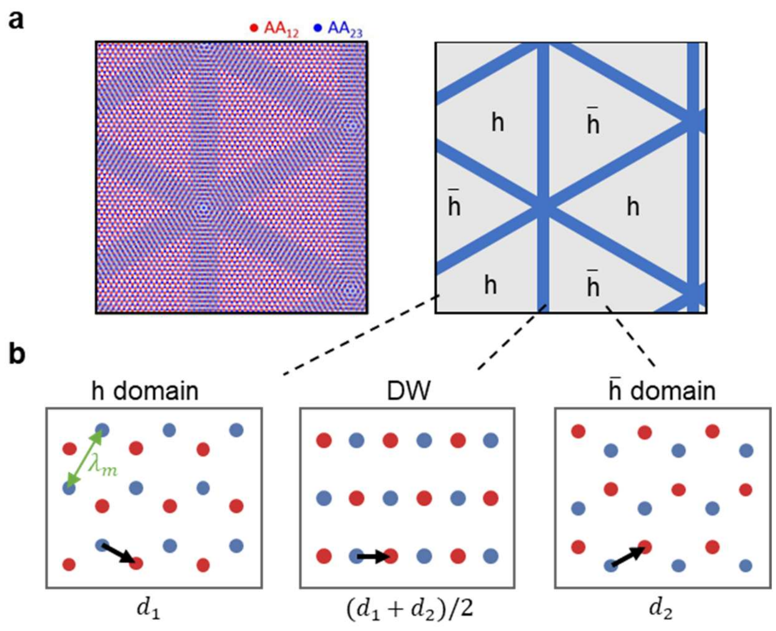

Fig. S13. Lattice configurations and domain structures of hTG.
(**a**) Simulated lattice configuration (left) and corresponding schematic representation (right). In the left panel, the red and blue dots represent the AA sites of two distinct moiré patterns. The right panel shows h-domain and $\bar{h}$-domain regions; the blue lines mark the domain boundaries. (**b**) Schematics of the local moiré configuration for each region. The domain regions are characterized by displacement vectors d, which represent the relative shift between the red and blue lattice point groups. The displacement vector of the domain wall (DW) region is defined as the average of those of the two adjacent domains.

In contrast to the periodic domains, the domain boundaries possess an aperiodic distribution of moiré sites due to moiré relaxation. For the band structure calculations, we focused on a representative part of the boundary region, referred to here as the domain wall (DW), which corresponds to the transitional area between two adjacent domains. This DW region was approximated using the average of the two adjacent displacement vectors, ($\mathbf{d}_1$+$\mathbf{d}_2$)/2, as illustrated in the middle panel of **Fig. S13b**.

The electronic structures of the h-domain and DWs, calculated for a single valley at a twist angle of 1.35° under this assumption, are shown in **Fig. S14a**, and the corresponding density of states (DOS) as a function of carrier density is presented in **Fig. S14b**. As indicated by the cyan arrows, both regions exhibit a suppression of DOS at $\nu = 0$ and ±4. This result supports our interpretation in **Fig. 3e** that the domain boundaries become resistive

at similar filling factors, consistent with the suppression of DOS. Notably, our calculations suggest the presence of valley-protected states in the DW region at $\nu = \pm 4$. If these dissipationless channels remain intact at the device scale, the measured resistance should be lower than the von Klitzing constant, h/e^2, which corresponds to a perfectly dissipationless channel. However, as shown in our data, the measured resistance at these fillings is significantly higher than this value, with some regions exceeding 100 kΩ, likely due to valley mixing induced by lattice disorder in the DW, which can open a gap.

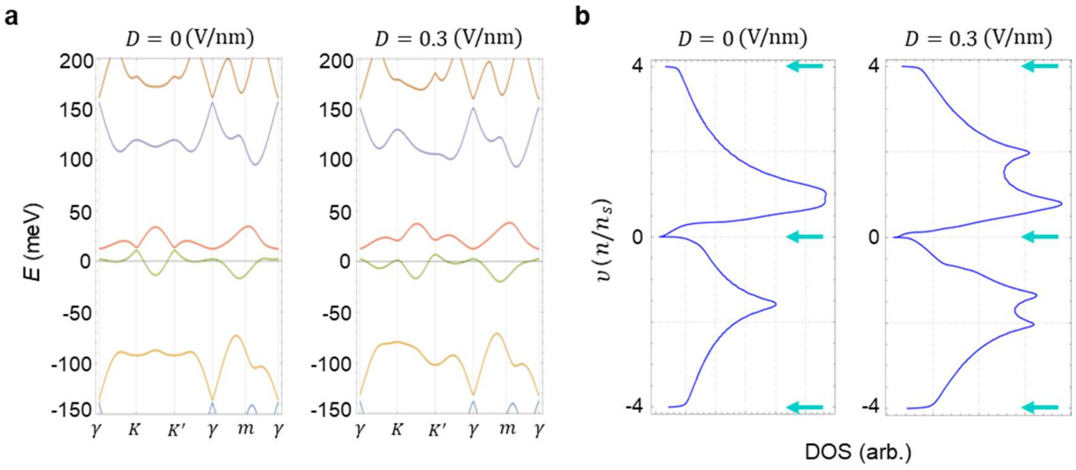

Fig. S14. Electronic band structures and density of states in the domain regions.
(**a**) Band structures calculated for the h-domain (left) and the DW region (right) for the K valley in a system with a twist angle of 1.35°. (**b**) DOS plots as a function of carrier density, derived from the band structure calculations, for the h-domain (left) and the DW region (right). The cyan arrows in each panel indicate the positions of ν = -4, 0, and 4, where the DOS is locally suppressed.

3.3. Band structure calculations at varying displacement fields

To examine the changes in the band structure of the domain regions under a D field, we varied the D values in our calculations and computed the corresponding electronic band structures for a single K valley, some of which are shown in **Fig. S15a**. When D = 0 V nm^{-1} (as shown in the first panel), the bands around the CNP are isolated and exhibit Chern numbers of +2 and -1, respectively. Notably, even when D = 0, each domain does not preserve C_{2z} symmetry and, as a result, can host nontrivial topology, which gives rise to these Chern numbers. As the D value increases, the gap between the two isolated bands around the CNP gradually decreases until they touch, as indicated by the pink circle in the second panel, and then increases again. Remarkably, as shown in the third panel, once the bands cross and separate their Chern numbers change from +2 and -1 to +1 and 0, confirming that this touching process represents a topological band inversion. The extracted band gap at the CNP during this process is plotted in **Fig. S15b**. The regions marked by pink arrows in the plot indicate where the two bands cross, which we associate with the local resistance minima observed in **Fig. 1c**.

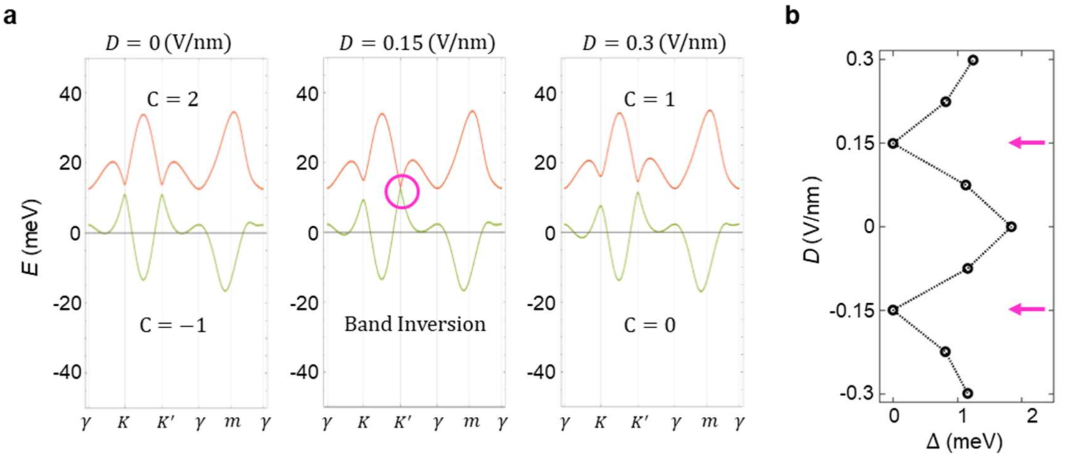

Fig. S15. Displacement field-dependent topological band inversion in hTG.
(**a**) Calculated band structures for the domain region at a twist angle of 1.35°, shown at varying displacement field values. The left, middle, and right panels correspond to $D = 0$, 0.15, and 0.3 V nm^{-1}, respectively. As D increases, the two bands near the CNP undergo a topological band inversion (highlighted by pink circle), resulting in a change in the Chern number of each band. (**b**) Band gap at the CNP as a function of D. The regions marked by pink arrows indicate the locations of topological band inversions.

Fig. S16 presents the calculated band structures over an extended energy range, in contrast to **Fig. S15**, which focuses on bands near the CNP. By expanding the vertical axis, additional higher-energy bands become visible, offering a broader view of the spectrum. Notably, sizeable band gaps—on the order of several tens of meV—remain open at filling factors $\nu = \pm 4$, even as the displacement field D is varied over a broad range from –0.6 to 0.6 V nm^{-1}. This persistence highlights the stability of the insulating states at these fillings against D field modulation.

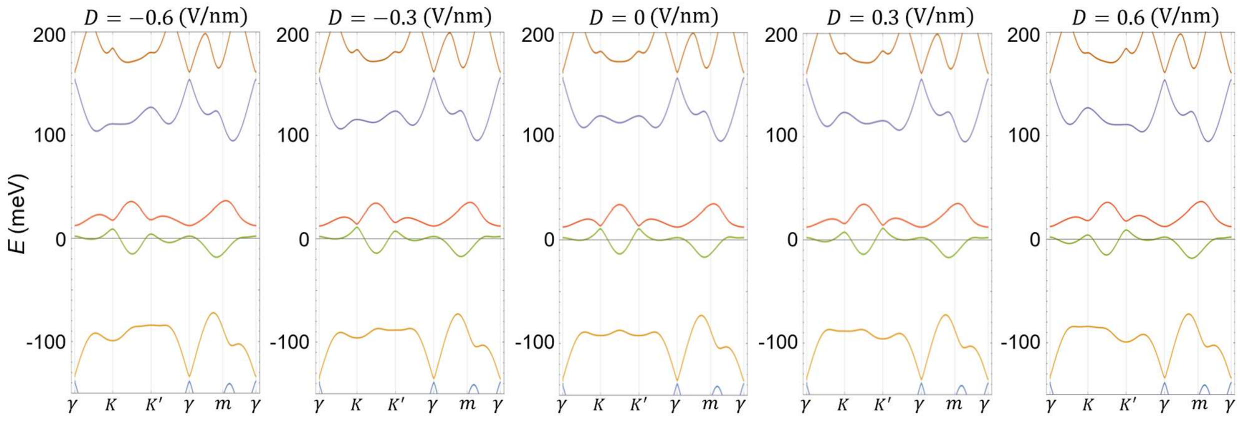

Fig. S16. Robustness of the large energy gaps across displacement fields.
Band structure calculations for a single domain region at a twist angle of 1.35°, shown for five different displacement field values: –0.6, –0.3, 0, 0.3 and 0.6 V nm^{-1} (from left to right). Each panel displays the electronic structure over an extended energy range.

4. Supplementary data on hysteretic transport behavior

This section provides supplementary data and interpretations related to the hysteretic transport behaviour of hTG, complementing the analysis presented in the main text.

4.1. Parallel resistance model interpretation of hysteresis

We model the total resistance of our hTG system in terms of its two constituent components—those from the domains and the domain boundaries—which are modelled as connected in parallel, as depicted schematically in **Fig. 2f**. As shown in **Fig. S17a**, the first panel (grey dashed line) schematically represents the resistance of the domains, R_{Domain}, and the second panel (blue dashed line) shows that of the domain boundaries, $R_{\mathrm{Boundaries}}$. These schematics are reconstructed based on the data presented in **Fig. 2**. Because $R_{\mathrm{Boundaries}}$ is regarded as exhibiting hysteretic behavior, its resistance peak can shift depending on the direction of the gate voltage sweep—this possibility is indicated by an arrow in the second panel. Under the assumption of a parallel configuration, the total resistance is given by

$$1/R_{\mathrm{total}} = 1/R_{\mathrm{Domain}}+1/R_{\mathrm{Boundaries}}.$$

The two rightmost panels of **Fig. S17a** show the resulting R_{total}. traces (red solid lines) for two distinct cases of $R_{\mathrm{Boundaries}}$, calculated using this relation. As seen in the third panel, the resulting resistance trace closely resembles the line-cuts in **Fig. 2g**, suggesting that the data can be naturally understood within this parallel configuration model. Conversely, with a measured R_{total}, one can use this model to estimate the two constituent resistances. In the main text, we applied this approach by assuming a maximum peak value of 1000 kΩ for $R_{\mathrm{Boundaries}}$, used as a free parameter required for separating the resistance components.

Additional support for this model is provided by the situation in which the resistive packet aligns with the dips in the non-hysteretic domain resistance. Under these circumstances, the model predicts that the packet should split around the band inversion point, as highlighted by the purple arrows in the rightmost panel of **Fig. S17a**. This prediction is verified by experiment: when the gate voltage is tuned such that the resistive packet coincides with one of the band inversions, it indeed separates into two smaller packets, as shown in the resistance map in **Fig. S17b**. The corresponding line-cut, presented in **Fig. S17c**, also exhibits a shape closely resembling the schematic in the rightmost panel of **Fig. S17a**, thereby reinforcing the validity of our model.

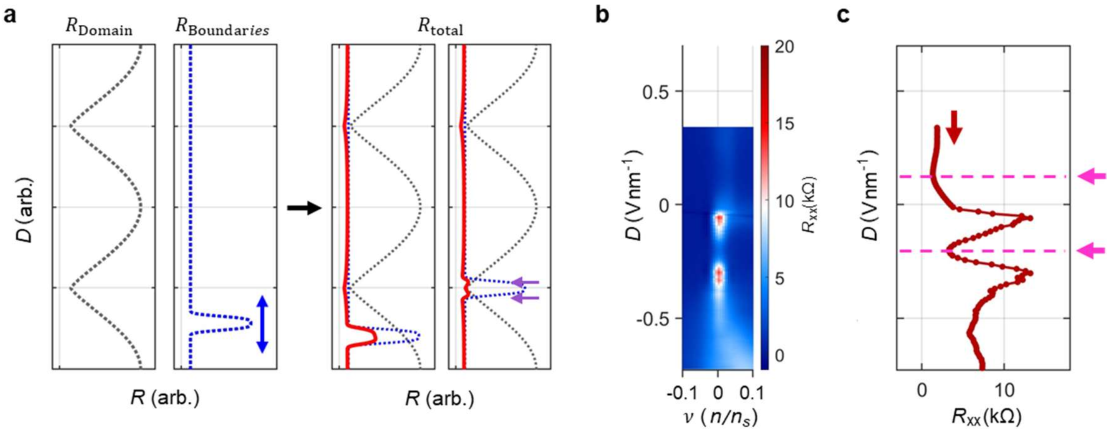

Fig. S17. Schematic of the parallel resistance model for hysteretic transport.
(**a**) Schematic illustrations of the resistance responses to varying D fields within different regions of the hTG system. The leftmost panel shows the resistance of the domain region (R_{Domain}, grey dotted line), and the second panel shows that of the domain boundaries ($R_{\text{Boundaries}}$, blue dotted line) with an arrow indicating the potential shift in resistance depending on the sweep direction. The two rightmost panels display the total resistance (R_{total}, red solid line), calculated by combining the two components in parallel according to the relation $1/R_{\text{total}} = 1/R_{\text{Domain}}+1/R_{\text{Boundaries}}$, for two different cases, with the individual components also shown in the same panels for comparison. (**b**) Longitudinal resistance map when the resistive packet is positioned at the band inversion point. (**c**) Maximum resistance value at each D field, extracted from b. The observed splitting of the resistive packet at the position marked by the pink arrow is consistent with the model prediction shown in (a).

4.2. Evolution of the resistive packet position in the *n*-*D* plane

In the main text, we highlighted that the Hall resistance (R_{xy}) not only exhibits hysteresis but also deviates from the $\nu = 0$ line. In contrast, the resistive packet is found to evolve along the $\nu = 0$ trajectory, as demonstrated in the data presented here. **Fig. S18a** shows R_{xx} maps acquired simultaneously with the R_{xy} maps presented in **Fig. 3** of the main text. The left and right panels correspond to upward and downward gate sweeps, respectively, with blue arrows indicating the sweep direction. All maps were measured at 4 K and ±0.2 T, and a pink dotted line is overlaid to indicate the $\nu = 0$ trajectory. In these maps, the high-resistance features near the CNP do not follow this line and instead appear slightly shifted with a finite slope. The resistive packet itself, however, consistently follows the $\nu = 0$ line. To further examine this behavior, we additionally acquired R_{xx} maps while varying the gate sweep range, as shown in **Fig. S18b**. These measurements were taken at 4 K and ±0.1 T. Blue arrows in each panel indicate both the direction of the sweep and the initial D field value. Each map again includes a pink dotted line representing the $\nu = 0$ position. Across all conditions, the high-resistance features near the CNP remain slightly deviated from the pink dotted line, yet the resistive packet continues to appear along it. These results confirm that the resistive packet consistently emerges along the $\nu = 0$ line.

This behavior is consistent with our interpretation of the hysteresis presented in **Fig. 3** of the main text. The non-hysteretic domains have their CNP aligned with $\nu = 0$, and the resistive packet arises when the CNP of the

hysteretic domain boundaries intersects this trace. Consequently, the packet should appear along the $\nu = 0$ line. The apparent deviation of the high-resistance features near the CNP can also be understood in this context: when the CNP of the domain boundaries deviates from $\nu = 0$, the high-resistance feature in the total resistance appears between the CNP positions of the domains and the domain boundaries, and thus deviates from the $\nu = 0$ line as well.

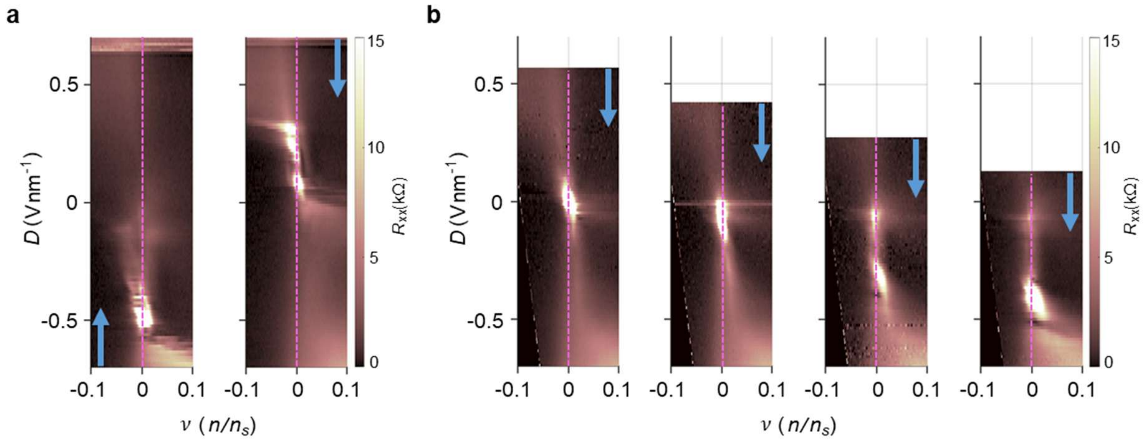

Fig. S18. Hysteretic evolution of the resistive packet position at the CNP.
(**a**)–(**b**) Longitudinal resistance maps plotted in the *n*–*D* plane near the CNP, measured at 4 K. Data in panels (a) and (b) were acquired under magnetic fields of ±0.2 T and ±0.1 T, respectively. Blue arrows indicate the gate sweep direction and the initial *D* field value for each sweep. Pink dashed lines mark the $\nu = 0$ position in all maps.

4.3. Hall resistance behavior at ±4 filling

While the main text focused on the hysteretic Hall response near the CNP (**Fig. 3a**), similar behavior is also observed near $\nu = \pm 4$. **Fig. S19a** and **b** shows R_{xy} maps near $\nu = -4$ and $+4$, respectively, measured at 4 K. Each display upward (left) and downward (right) gate sweeps, with pronounced sweep-direction-dependent responses analogous to those at the CNP. However, due to the highly resistive features at exactly $\nu = \pm 4$, the Hall signals are too noisy to reliably extract the carrier density of the domain boundaries using the method employed in the main text. We therefore analyzed line cuts slightly offset from these fillings—specifically at $\nu = -3.973$ and $+3.973$. The longitudinal resistance along these lines reveals regions with high resistance (exceeding 100 kΩ), indicating that the domain remains highly resistive along these trajectories. Therefore, consistent with the analysis presented in Supplementary Section 5, the Hall resistance along these lines can still be used to estimate the carrier density in the domain boundaries. The extracted carrier densities at each filling are summarized in **Fig. 3e**.

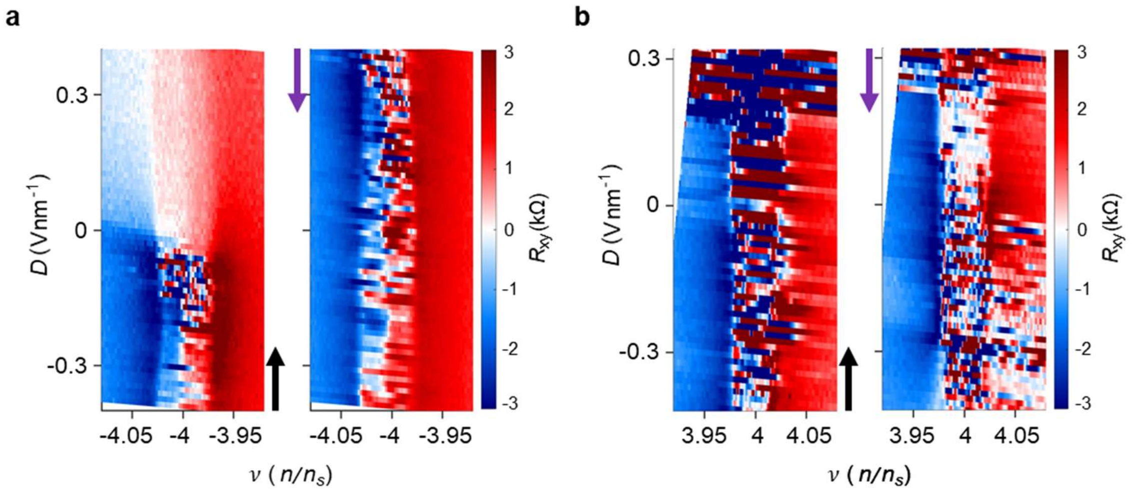

Fig. S19. Hysteretic Hall resistance near $\nu=\pm4$.
(**a**)–(**b**) Hall resistance maps plotted in the *n*–*D* plane near ν = -4 (a) and ν = +4 (b), each showing measurements taken during upward (left) and downward (right) gate sweeps. All data were acquired at 4 K. These datasets reveal pronounced hysteretic behavior, analogous to that observed near the CNP.

5. Finite-element analysis of Hall response

To investigate the Hall response in the hTG system containing both domains and domain boundaries, we carried out finite-element simulations using the COMSOL Multiphysics software. The calculations were performed using the 2D Electrostatics interface in stationary mode. We modelled a representative geometry consisting of a periodic array of domains and domain walls, confined to a rectangular region of 12 μm in length and 3 μm in width, as shown in **Fig. S20a**. Within this area, the supermoiré periodicity is 450 nm and the domain wall width is 65 nm. The chosen moiré length of 450 nm roughly corresponds to that of hTG with a twist angle of ~1.35°.

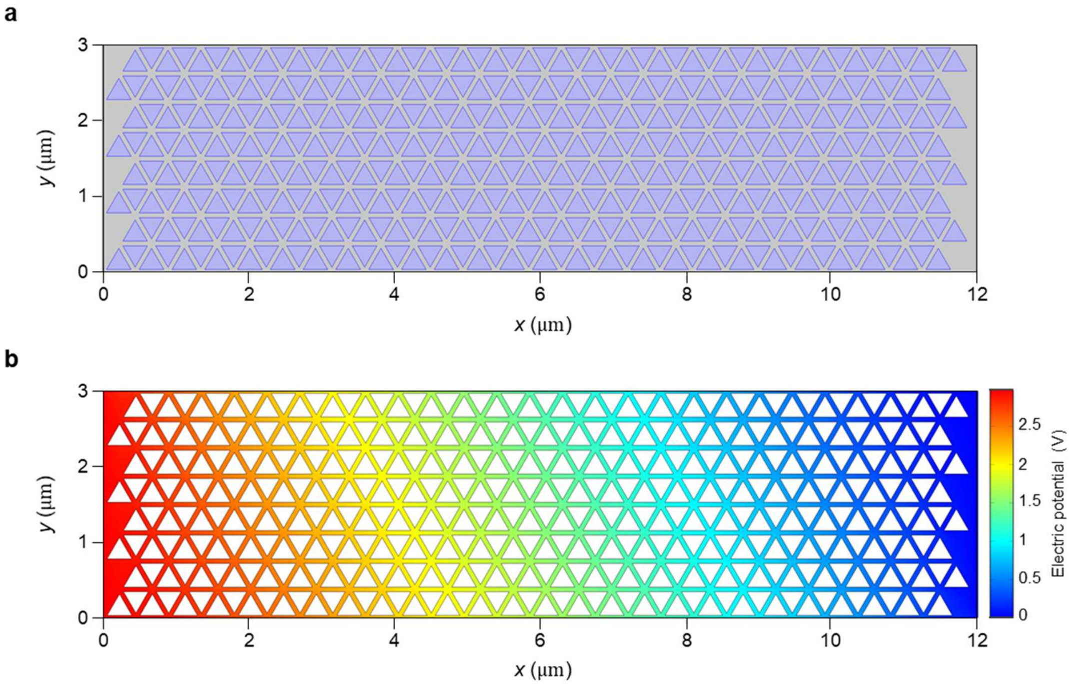

Fig. S20. Finite-element analysis of the Hall effect in the domain boundary network.
(**a**) Geometry used for COMSOL simulation, consisting of a periodic array of triangular domains (purple) embedded in a background representing the domain boundaries (grey). (**b**) Simulated spatial distribution of the electric potential, visualized only within the domain boundaries, under a 1 A current applied from left to right. The conductivity tensor in the domain boundaries is set to σ = [1 2; –2 1], corresponding to μB = 2, while the domains have a conductivity 10^{-5} times lower.

For the boundary conditions, a constant current of 1 A was applied along the left edge ($x = 0$) while the right edge ($x = 12$ μm) was grounded, maintaining an equipotential surface on the current injection side. To emulate the effect of an out-of-plane magnetic field, we introduced an off-diagonal term in the conductivity tensor. In the domain boundaries, the conductivity tensor was set to σ = [1 2; -2 1], based on an assumed mobility of 100,000 cm^2/Vs under a magnetic field of 0.2 T, such that μB = 2. In the initial setup, the domains were treated as highly insulating regions, with their conductivity set to 10^{-5} times that of the boundaries. For simplicity, we set the

reference conductivity $\sigma_0 = 1$. These parameter values were set for illustrative purposes, as the resulting potential profile depends only on their relative scales. **Fig. S20b** shows the resulting spatial distribution of the electric potential, visualized only within the domain boundaries. The calculated potential, despite being limited to the domain boundaries, appears qualitatively similar to that of a homogeneous system.

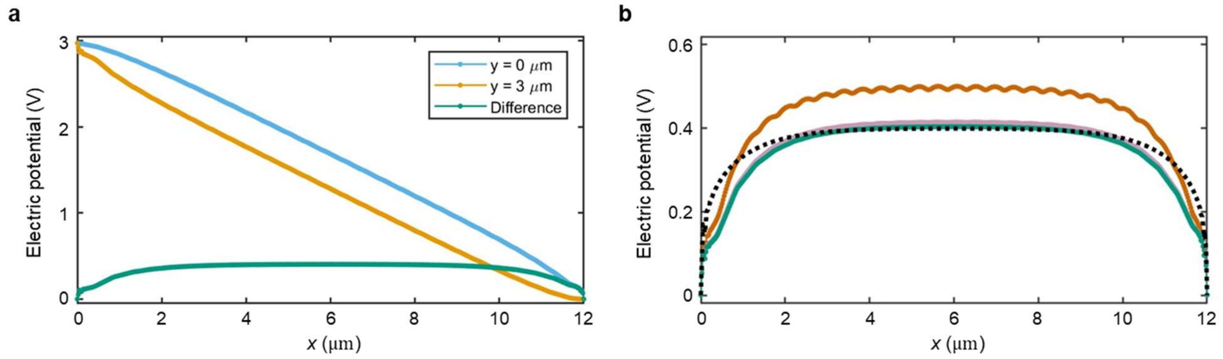

Fig. S21. Simulated Hall voltage and its dependence on domain conductivity contrast.
(**a**) Electric potential along the bottom ($y = 0$ μm, light blue) and top ($y = 3$ μm, orange) edges of the simulated geometry, and their difference (green), corresponding to the Hall voltage. The potential difference remains nearly constant in the central region, away from the sample edges. (**b**) Calculated Hall voltages for different conductivity ratios between the domains and the domain boundaries: 0.1 (brown), 0.01 (lavender grey), and 10^{-5} (green, same as in panel (a)). The black dashed line corresponds to the result for a homogeneous system without domain structure.

Since the Hall voltage is defined as the potential difference across the edges perpendicular to the current flow, we examined it by extracting the electric potential along the top ($y = 3$ μm) and bottom ($y = 0$ μm) edges of the simulated region in **Fig. S20b**. As shown in **Fig. S21a**, the light blue and orange traces represent the potential along $y = 0$ and $y = 3$ μm, respectively, and their difference is shown in green. The potential difference remains nearly constant across the central part of the sample, away from the edges, corresponding to the Hall voltage that would be measured experimentally.

To understand how this Hall voltage varies with the conductivity of the domain regions, we performed additional simulations while varying the conductivity ratio between the domains and the domain boundaries. **Fig. S21b** shows the resulting potential differences for three cases: a ratio of 0.1 (brown), 0.01 (lavender grey), and 0.00001 (green, same as in **Fig. S21a**). The black dashed line represents the result from a homogeneous geometry with no domain structure. As the domains become more insulating, the calculated Hall voltage converges to that of the homogeneous case. This suggests that when the domains are sufficiently resistive, the inhomogeneous geometry has minimal influence on the measured Hall voltage. In such cases, the Hall voltage continues to accurately reflect the carrier density as in a uniform system, following the relation $n = B/(eR_{xy})$.

6. Discussion on the aperiodic moiré structure as an effective disorder

The hTG system naturally forms domain regions composed of commensurate moiré sites and domain boundaries with aperiodic structures through moiré relaxation. This structural contrast is clearly illustrated in the simulation result of **Fig. S22a**, which displays both periodic and aperiodic arrangements of moiré sites. In this panel, the left and right regions exhibit periodic arrangements, where the red and blue dots—representing the AA sites of two distinct moiré patterns—form a honeycomb-like structure. In contrast, around the $x \approx$ -50 nm region, these moiré sites become deformed, deviating from the periodic order.

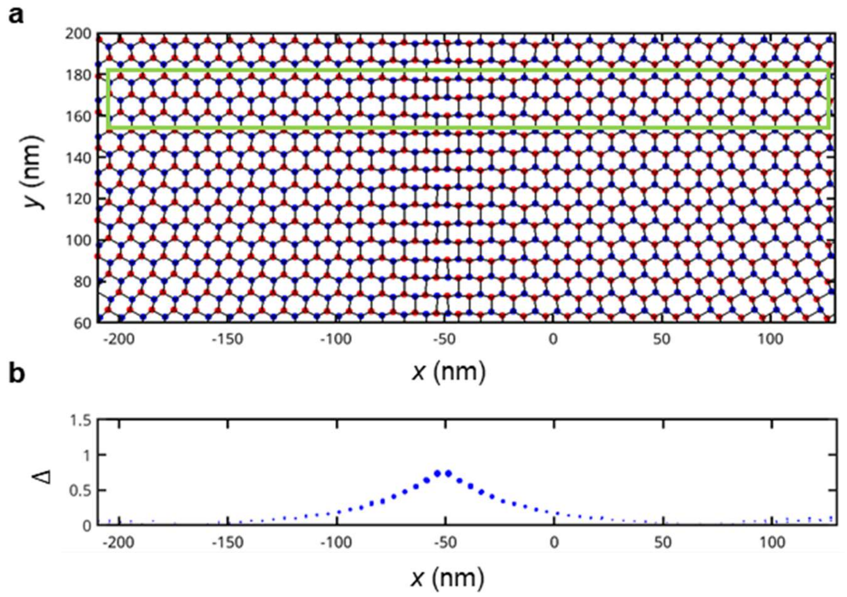

Fig. S22. Moiré relaxation and concentrated structural disorder at domain boundaries.
(**a**) Moiré relaxation simulation results for hTG with a twist angle of 1.4°. The red and blue dots indicate the AA sites of two distinct moiré potentials: one originating from the top and middle layers, and the other from the middle and bottom layers. For visual clarity, black solid lines are drawn between adjacent dots of different color. Around the x = -50 nm region, a domain boundary emerges where the moiré potential sites become aperiodic. (**b**) Lattice deformation factor plotted along the x-axis. Within the commensurate domain regions, the factor remains close to zero, whereas it increases sharply near the domain boundary.

Within the domain regions, the periodic moiré sites act as a long-range potential, renormalizing the electronic states in the low-energy band. In this context, the aperiodic moiré sites in the domain boundaries can be regarded as an additional perturbation to the periodic moiré potential, which in turn implies that this aperiodicity may act as an *effective* disorder. To characterize the degree of this effective disorder, we introduce the lattice deformation factor, Δ, defined as:

$$\Delta = |\Sigma_{i=1,2,3}\, a_i| \,/\, \sqrt{(\Sigma_{i=1,2,3}|a_i|^2)}$$

For each moiré site, we compute the sum of displacement vectors to its three nearest neighbors. Δ is then obtained by normalizing this sum by the square root of the sum of squared vector magnitudes. This value is zero when the moiré sites are periodically arranged in a honeycomb structure, but it increases as the structure deviates from this periodicity. **Fig. S22b** shows the lattice deformation factor Δ plotted along the x-axis from our simulation. As expected, Δ remains close to zero within the domain regions, while it increases significantly near the domain boundary. These results support the idea that the aperiodic structure at the boundaries introduces an effective disorder. Given this, it is plausible that such disorder could lead to strong localization of electronic states that would otherwise remain extended.

References

1. X. Lu, B. Lian, G. Chaudhary, B. A. Piot, G. Romagnoli, K. Watanabe, T. Taniguchi, M. Poggio, A. H. MacDonald, B. A. Bernevig, and D. K. Efetov, Multiple flat bands and topological Hofstadter butterfly in twisted bilayer graphene close to the second magic angle. Proc. Natl. Acad. Sci. U.S.A. **118**, e2100006118 (2021).
2. Y. Jeong, H. Park, T. Kim, K. Watanabe, T. Taniguchi, J. Jung, and J. Jang, Interplay of valley, layer and band topology towards interacting quantum phases in moiré bilayer graphene. Nat. Commun. **15**, 6351 (2024).
3. J. M. Park, Y. Cao, K. Watanabe, T. Taniguchi, and P. Jarillo-Herrero, Tunable strongly coupled superconductivity in magic-angle twisted trilayer graphene. Nature **590**, 249 (2021).
4. Z. Hao, A. M. Zimmerman, P. Ledwith, E. Khalaf, D. H. Najafabadi, K. Watanabe, T. Taniguchi, A. Vishwanath, and P. Kim, Electric field–tunable superconductivity in alternating-twist magic-angle trilayer graphene. Science **371**, 1133 (2021).
5. T. Devakul, P. J. Ledwith, L.-Q. Xia, A. Uri, S. C. de la Barrera, P. Jarillo-Herrero, and L. Fu, Magic-angle helical trilayer graphene. Sci. Adv. **9**, eadi6063 (2023).
6. J. C. Hoke, Y. Li, Y. Hu, J. May-Mann, K. Watanabe, T. Taniguchi, T. Devakul, and B. E. Feldman, Imaging supermoire relaxation and conductive domain walls in helical trilayer graphene. Preprint at https://doi.org/10.48550/arXiv.2410.16269 (2024).
7. L.-Q. Xia, S. C. de la Barrera, A. Uri, A. Sharpe, Y. H. Kwan, Z. Zhu, K. Watanabe, T. Taniguchi, D. Goldhaber-Gordon, L. Fu, T. Devakul, and P. Jarillo-Herrero, Topological bands and correlated states in helical trilayer graphene. Nat. Phys. **21**, 239 (2025).
8. J. Jang, Effect of lattice relaxation on electronic spectra of helically twisted trilayer graphene: large-scale atomistic simulation approach. J. Korean Phys. Soc. **85**, 727 (2024).
